# Are Cyanobacteria an Ancestor of Chloroplasts or Just One of the Gene Donors for Plants and Algae?

**DOI:** 10.3390/genes12060823

**Published:** 2021-05-27

**Authors:** Naoki Sato

**Affiliations:** Graduate School of Arts and Sciences, University of Tokyo, Meguro-ku, Tokyo 153-8902, Japan; naokisat@bio.c.u-tokyo.ac.jp

**Keywords:** chloroplast origin, cyanobacterial endosymbiosis, glycolipids, host-directed chloroplast formation, peptidoglycan, *Paulinella chromatophore*, phylogenetic analysis

## Abstract

Chloroplasts of plants and algae are currently believed to originate from a cyanobacterial endosymbiont, mainly based on the shared proteins involved in the oxygenic photosynthesis and gene expression system. The phylogenetic relationship between the chloroplast and cyanobacterial genomes was important evidence for the notion that chloroplasts originated from cyanobacterial endosymbiosis. However, studies in the post-genomic era revealed that various substances (glycolipids, peptidoglycan, etc.) shared by cyanobacteria and chloroplasts are synthesized by different pathways or phylogenetically unrelated enzymes. Membranes and genomes are essential components of a cell (or an organelle), but the origins of these turned out to be different. Besides, phylogenetic trees of chloroplast-encoded genes suggest an alternative possibility that chloroplast genes could be acquired from at least three different lineages of cyanobacteria. We have to seriously examine that the chloroplast genome might be chimeric due to various independent gene flows from cyanobacteria. Chloroplast formation could be more complex than a single event of cyanobacterial endosymbiosis. I present the “host-directed chloroplast formation” hypothesis, in which the eukaryotic host cell that had acquired glycolipid synthesis genes as an adaptation to phosphate limitation facilitated chloroplast formation by providing glycolipid-based membranes (pre-adaptation). The origins of the membranes and the genome could be different, and the origin of the genome could be complex.

## 1. Introduction

Cyanobacteria are a distinct group of bacteria that perform oxygenic photosynthesis. They have specialized internal membranes called thylakoid membranes in which photosynthetic systems reside. Galactolipids and sulfolipids that are major constituents of the thylakoid membranes have been believed to be necessary for the photosynthetic activity. Cyanobacteria and chloroplasts of plants and algae share thylakoid membranes with galactolipids and sulfolipids, various components of photosystems, and photosynthetic pigments. These traits of substance, structure, and function as a whole were taken as good evidence for the cyanobacterial origin of chloroplasts. The endosymbiotic hypothesis of chloroplast origin was first proposed by C. Mereschkowsky in 1905 [1] who obtained a hint from a short note of A. Schimper in 1883 [2]. Since then, it remained a well-known but unsupported hypothesis. Various authors in the 1960s revitalized the hypothesis based on the discovery of chloroplast DNA, and the similarities of chloroplasts and cyanobacteria became evident thanks to the developments in cell biology and biochemistry of photosynthetic organisms in the 1960s and 1970s. It was the phylogenetic analysis in the 1980s and later that provided convincing evidence for the cyanobacterial origin of chloroplasts. Historical views on the endosymbiotic origin of chloroplasts are explained in some review articles [3,4,5,6,7,8,9,10].

All textbooks of biology explain the origin of chloroplasts by cyanobacterial endosymbiosis that occurred 1 or 2 billion years ago. The origin of mitochondria is also explained by the endosymbiosis of a proteobacterium. These origin stories are currently considered as well-established facts that form the basis of the formation of eukaryotic cells. Many phenomena of eukaryotic cells are currently understood in terms of the endosymbiotic origin of organelles. Stiller [11] criticized this situation: “While many research articles and reviews refer to a single primary origin of all plastids as an established fact, it remains a working hypothesis that gained popularity under a set of assumptions that turn out to have little empirical support in nature (p. 467)”.

There are still two major questions about the endosymbiotic theory or hypothesis. First, what is exactly the decisive evidence that the chloroplasts and mitochondria originated from cyanobacterial and proteobacterial endosymbionts, respectively, and how and when this theory was established? It is well known that, in the late 1960s and 1970s, Lynn Margulis claimed her original version of the origin of eukaryotic cells, which involved the endosymbiotic origin of chloroplasts, mitochondria, and mitotic apparatus or flagella [12,13]. Nevertheless, it was the molecular phylogeneticists that presented convincing evidence for the close relationship between chloroplasts and cyanobacteria, and between mitochondria and α-proteobacteria. The accumulation of supporting data continued until the early 2000s when the genomic sequences of major model organisms became available. Under these situations, not only the organellar genes but also nuclear-encoded organelle proteins were used for the phylogenetic analysis that finally confirmed the close relationships, chloroplasts/cyanobacteria, and mitochondria/α-proteobacteria. Nevertheless, this is just a story of organellar DNA. DNA origin is not enough to affirm the two endosymbiotic events as illustrated in many books and review articles. Membranes and genomes are essential components of a cell or an organelle such as chloroplasts and mitochondria. Can we deduce an endosymbiotic image of the origin of an organelle directly from its DNA origin?

Another question, related to this point, arises from more recent studies on the extensive phylogenetic studies on many enzymes involved in the characteristic, common traits of chloroplasts and cyanobacteria. Most of the enzymes involved in the synthesis of thylakoid membrane lipids in plants and algae do not originate from cyanobacteria. How can we explain this? In mitochondria, systematic phylogenetic analysis of the full complement of mitochondrial proteins also raised a comparable question: namely, how can we explain that 90% of mitochondrial enzymes [14,15] existed in the eukaryotic cell long before the formation of mitochondria? Keeling [16] pointed out the “cognitive inertia” of endosymbiotic thinking, and suggested removing the historically biased view: “It is interesting, therefore, to consider what kinds of conclusions we might draw if we learned what we know today in a different order, or learned it all at once (p. 10)”. It is worth trying to re-examine all available evidence for the endosymbiotic theory and to re-organize the history of the formation of chloroplasts and mitochondria. Therefore, this is a discussion or hypothesis article with a limited length on the historical and biological questions on the endosymbiotic origins of organelles, but I focus mainly on the relationship between cyanobacteria and chloroplasts.

Because many readers in biology might not be familiar with this kind of half-philosophy, half-biology article, I first present the overall plan of the article. There are three major, mutually related topics: (1) History section (Section 2) deals with the ambiguous meaning of “endosymbiosis”. Endosymbiosis notion includes physical integration of cyanobacterial cell into a eukaryotic cell, but this cannot be proven by cytological experiments. Only DNA origin can be identified by phylogenetic analysis. Then, I discuss the visually identifiable components (membranes) and genome in the following two sections. (2) The lipid and peptidoglycan section (Section 3) deals with the problem that the structural elements of chloroplasts did not originate from cyanobacteria. (3) Chloroplast gene section (Section 4) deals with putative, multiple origins of chloroplast genome within cyanobacteria. Indeed, this is mostly a re-interpretation of previous results. Section 5 also discusses the origins of some other chloroplast enzymes. After these three parts, Section 6 is devoted to discussing the *Paulinella* model of chloroplast formation, and I present a role of pre-adaptation that determined the fate of chloroplasts. This is not a simple rejection of the endosymbiotic hypothesis, but a broadening of the hypothesis. I also have to mention that the topic of the present article is limited to the formation of “primary” chloroplast. The secondary endosymbiosis must be a different story.

## 2. Historical and Theoretical Problems on the Origin of Chloroplasts

### 2.1. Historical Demarcation

The relationship between cyanobacteria and chloroplasts has been repeatedly argued since Mereschkowsky [1] (see also an English translation [3]). I already presented a detailed historical analysis of the endosymbiotic theories [8,9]. Readers are referred to these as well as [4,5,6,17] for the history of endosymbiotic theories. In the early 20th century, the similarity of cyanobacteria and chloroplasts was restricted to the similarity in size, the presence of photosynthetic pigments, and the activity of carbon fixation with oxygen evolution (“photosynthetic oxygen production” has been conventionally called “oxygen evolution”), all of which reflected the status of knowledge at the time. The history of endosymbiotic arguments may be divided into three major phases: In the first phase (1905–1961), the similarity of cyanobacteria and chloroplasts in terms of photosynthetic properties as defined above was recognized by many researchers in plant and algal studies, but the majority of top researchers, such as Pascher [18] and Buchner [19], clearly rejected endosymbiotic origin of any eukaryotic organelle (chloroplasts or mitochondria). However, Pascher [18] distinguished cyanelles from chloroplasts and described that the cyanelles of *Cyanophora paradoxa* and *Paulinella chromatophora* were authentic cyanobacterial cells living in a eukaryotic cell. Cyanelles were evidence of the cyanobacterial origin of chloroplasts for some researchers but were distinct from chloroplasts for many others. Lederberg [20] suggested later that the genetic system supposed to reside in chloroplasts was a kind of plasmid, an exogenous genetic element. Symbiogenesis was also supported by some speculative biologists or philosophers [21,22].

In the second phase (1962–1977), the presence of organellar DNA in chloroplasts and mitochondria was taken as evidence for the bacterial origin of these organelles. In parallel, cytological and biochemical characterization of chloroplasts and mitochondria enabled precise comparison of these organelles with cyanobacteria and other aerobic bacteria. The prokaryotic nature of the organellar ribosomes (70S sedimentation-based size, partial rRNA sequence, sensitivity to antibiotics) was an important, novel fact that supported the similarity of organelles and bacteria. Moreover, physical characterization of photosynthetic mechanisms, biochemical identification of photosystem components, and chemical analysis of lipid components of thylakoid membranes, all these data were additional evidence for the similarity of chloroplasts and cyanobacteria. In 1966, Echlin presented a comprehensive table comparing cyanobacteria and chloroplasts [23]. Hybridization of chloroplast rRNA and cyanobacterial DNA was also innovative molecular evidence that favored the similarity of cyanobacteria and chloroplasts. Nevertheless, all these similarities could be used in both ways, namely, for and against the endosymbiotic origin of chloroplasts. In 1967, Goksøyr published a short paper in *Nature*, illustrating the sequential endosymbiotic origins of mitochondria and chloroplasts [24] (note that this preceded the 1967 paper of Lynn Margulis (Lynn Sagan then) [12], and the Serial Endosymbiosis Theory (SET) of Taylor published in 1974 [25]). The autogenous origin of chloroplasts could also explain the data [26,27,28], although it was difficult to explain the origin of both chloroplasts and mitochondria within a single scheme. Prudent opinions remained, while changes began to emerge [29,30,31,32].

The third phase was marked by the development of phylogenetic analysis, which was the breakthrough that changed the status of the endosymbiotic theory. An epoch-making paper of Schwartz and Dayhoff appeared in 1978, reporting a reliable phylogenetic relationship between cyanobacteria and chloroplasts [33]. Phylogenetic analysis was indeed the only rational way of identifying the direct lineage from cyanobacteria to chloroplasts. The methodology of phylogenetic inference became more and more accurate and reliable. Innovations in both computer hardware and computational technology, and dramatic speedup in DNA sequencing and assembly helped the progress of phylogenetic analysis. From the 1980s onwards, chloroplasts became regarded as descendants of cyanobacteria. Nevertheless, it is difficult to distinguish when the conceptual change occurred. The change seemed to proceed gradually and slowly during the 1990s and 2000s. Phylogenetic data accumulated, and all data tended to be interpreted in the light of the endosymbiotic theory, namely, the cyanobacterial origin of chloroplasts. Then, no other way of thinking became mentioned in the literature.

### 2.2. Genocentrism and the Changes in Thinking at the Beginning of the 21st Century

I have to point out curious changes in the thinking of researchers from the 1980s to the early 2000s regarding the endosymbiotic origin of organelles, especially the cyanobacterial origin of chloroplasts. The review article of Gray and Doolittle [34] affirmed the cyanobacterial origin of chloroplasts, based on various phylogenetic trees. Among them, the most important was the phylogenetic tree of rRNA, because the ribosome is the core of the gene expression system in any organism. Wallace [35] also discussed the origin of organelle genomes and concluded:

“All of these data are consistent with the hypothesis that the chlDNA genes diverged from the blue-green algae well after the divergence of the eubacterial and eucaryotic nuclear lines. Therefore, chlDNAs must have originated by a symbiotic event between members of the eubacteria and eucaryotic nuclear lineages. Similarly, sequence data have indicated that the mtDNA rRNA genes are more homologous to bacterial rRNA genes than to rRNA genes of eukaryotic nuclei. They too probably also had an endosymbiotic origin.” ([35] p. 227)

The origin of organelle DNA was considered convincing evidence for the endosymbiotic origin of organelles because no other way of acquiring exogenous DNA was known in the 1980s. The notion of horizontal gene transfer appeared later, but the phylogenetic data supporting endosymbiosis were no longer examined in the light of horizontal gene transfer. Delwiche et al. [36] reported the results of robust phylogenetic analyses of the *tufA* gene, and concluded: “The finding from *tufA* that all plastids are derived from cyanobacteria is compatible with analyses of 16S rRNA and *atpB* sequences” (p. 124). Moreira and Philippe [37] wrote in 2001: “a wealth of data strongly suggests that all known photosynthetic plastids are monophyletic and that they have a cyanobacterial origin” (p. 771) by citing Delwiche [38], who, nevertheless, noted cautiously in the legend for the illustration of endosymbiotic origin of plastids: “One hypothesis for endosymbiotic events in the evolution of plastids. … This figure assumes a single origin of plastids, but this remains uncertain, as does the precise relationship among the primary plastid lineages” (p. S168). Looking back at the literature, the beginning of the 21st century was a turning point, in which the cyanobacterial origin of chloroplasts became accepted as almost established, although the debate on the monophyly of primary plastids continued [39,40]. Certainly, the genome sequencing of the cyanobacterium *Synechocystis* sp. PCC 6803 [41] and other cyanobacteria opened a way of directly comparing the genomes of cyanobacteria and chloroplasts, and the accumulation of sequence data of both cyanobacteria and chloroplasts facilitated the comparison of the two entities. McFadden [42] listed various lines of convincing evidence, and wrote also in 2001: “the endosymbiotic origin of plastids from cyanobacterial ancestors thus seems confirmed.” (p. 953)

The similarity of RNA polymerase in cyanobacteria and chloroplasts was also known in the 1990s. As described in biochemistry textbooks, bacterial RNA polymerases consist of α, β, β’, and σ subunits. However, in the cyanobacterial counterpart, the β’ subunit is split into two parts, called β’ and β’’. This split is found at the level of the gene, as well as protein. This strange situation is also found in RNA polymerases encoded by the chloroplast genome (see the references cited in [36] for the genes that served as evidence for the cyanobacterial origin of plastids). Essential components of gene expression in the chloroplast were found almost cyanobacterial. The chloroplast genome was also found to be similar to the cyanobacterial genomes, except for the large difference in sizes. Almost all enzymes and RNA that are encoded by the chloroplast genome had clearly identifiable cyanobacterial counterparts. The similarity of photosynthetic proteins was evident: the components encoded by the chloroplast genome had orthologs in cyanobacteria. However, in many cases, the photosynthesis-related components were only found in cyanobacteria and chloroplasts, and it was not possible to identify the root; namely, it was not possible to find where the evolution started. Another problem was unidentified DNA polymerase (see Section 5.1) in chloroplasts. Nevertheless, the majority of opinions favored the cyanobacterial origin of chloroplasts.

In retrospect, this was certainly motivated by the importance of genome and gene expression systems in biology. The cyanobacterial nature of the genetic apparatus of chloroplasts including DNA, RNA polymerase, and ribosomal components was taken as evidence that the chloroplast was a descendant of cyanobacteria. I call this “genocentrism” (gene-centered view), which was certainly the decisive impetus that changed the view on the origin of chloroplasts. I have to point out, however, that the genocentrism has been hidden behind the illustrations clearly showing the membranes to explain the endosymbiotic origin of organelles in many review articles [5,6,43,44,45] and textbooks (see for example [46]). In contrast, only a vague relationship was known between the cyanobacterial membranes and the chloroplast membranes. The presence of galactolipids and sulfolipids, as well as various photosynthetic components, was known in both cyanobacteria and chloroplasts, which was the evidence for the similarity of thylakoid membranes. The origin of the chloroplast envelope remained ambiguous: it could originate from the cyanobacterial plasma membrane, modified endoplasmic reticulum, phagosome, or peri-algal vacuole. Any origin was possible.

### 2.3. Spread of Endosymbiotic Gene Transfers (EGT)

Many components of the chloroplast ribosome were found to be encoded by the nuclear genome. Phylogenetic analysis suggested that these nuclear genes originated from chloroplast genes. This was explained by the endosymbiotic gene transfer (EGT), namely, chloroplast genes were somehow transferred to the nucleus [47]. This was supported by artificial EGT experiments [48]. A fairly efficient gene flow from the chloroplast genome (and the mitochondrial genome) to the nucleus was supposed to work during the long history of plant evolution (for a review of the time, see [49]).

However, as the cases of EGT became abundant, many other nuclear genes encoding chloroplast proteins have been simply considered to result from EGT, such as the peptidoglycan synthesis enzymes that were identified in the nuclear genome of the moss *Physcomitrella patens*. Various other chloroplast enzymes were also identified in plant nuclear genomes. In many cases, phylogenetic analysis was not performed. In other cases, only cyanobacterial sequences and plant sequences were used in phylogenetic analysis. Bacterial sequences were rarely used to identify the origin of the nuclear genes.

### 2.4. Currently Accepted Evidence for the Endosymbiotic, Cyanobacterial Origin of Chloroplasts

Currently, several different lines of evidence for the cyanobacterial origin of chloroplasts exist (Table 1. See also [9,50]). Photosynthesis with oxygen evolution (Item 1) is the best trait that suggests a direct relationship between cyanobacteria and chloroplasts, which also have structural and biochemical similarities. Structurally, both have internal, flattened membranes called “thylakoid” (meaning a sac) membranes, which are the site of light-driven reactions of photosynthesis. The chloroplast is surrounded by a set of limiting membranes called envelope membranes, whereas the cyanobacterial cell is enclosed by inner and outer membranes. Note that *Gloeobacter* lacks thylakoid membranes, and photosynthetic reaction centers reside in the inner cell membrane. Additional and related shared traits in cyanobacteria and chloroplasts include the presence of photosynthetic pigments such as chlorophyll *a* and the presence of galactolipids in the membranes. The presence of DNA and its associated genetic system (Item 2) is evidence for the autonomy of chloroplasts, and therefore, evidence for the exogenous origin of chloroplasts, implying endosymbiotic origin. Reproduction by binary fission (Item 3) is another similarity between chloroplasts and cyanobacteria. Another classical evidence is the presence of peptidoglycan (Item 4) in some chloroplasts, which will be explained in detail later (Section 3.1). The presence of cyanelles, which were believed to be living cyanobacterial cells within a photosynthetic eukaryote (see Section 2.1), is no longer evidence for the visual cyanobacterial endosymbiosis that can explain chloroplasts origin. The cyanelle of *Cyanophora* is now recognized as a chloroplast, rather than a cyanobacterium. The cyanelle of *Paulinella* is not an independent organism, which will be discussed later.

Next, phylogenetic analysis of chloroplast-encoded genes (Item 5; see Section 4) showed their cyanobacterial origin. Nearly all protein-coding genes and RNA genes encoded by the chloroplast genomes of algae and plants originated from cyanobacteria. Rare exceptions will be explained in Section 4.

Another line of evidence is the synteny of related genes in the chloroplast genomes and the cyanobacterial genomes (Item 6) [51,52]. The order of gene arrangement in the ribosomal protein cluster is highly conserved in bacteria, and the same order is found in the chloroplast genome if missing genes are allowed. The missing ribosomal proteins are supposed to be in the nuclear genome as a result of EGT. This idea was often verified by subsequent genomic analyses. Synteny is also found in the genes coding for ATPase subunits and RNA polymerase subunits, although there are many supposedly missing genes. Synteny is considered superior to phylogenetic trees because the latter could be affected by calculation methods and data sets, but there is no doubt about the order of genes.

### 2.5. Archaeplastida and the Cyanobacterial Origin of Chloroplasts

Many researchers argue that the monophyletic origin of the three lineages, namely, green algae/plants, red algae, and glaucophytes, is an important piece of evidence for the cyanobacterial origin of chloroplasts. Phylogenetic analysis of chloroplast genes invariably shows (with some exceptions as already mentioned) that the chloroplast genes diverge from the cyanobacterial clade as a single clade [53,54]. This is taken as evidence for the cyanobacterial origin of chloroplasts. Besides, extensive phylogenetic analysis of the 143 nuclear genes supports the monophyly of the three lineages [55]. Additionally, we showed that the chimeric origins of carbon fixation cycle (also called Calvin-Benson cycle) enzymes are common in the red algae and the green plants [56], which was subsequently confirmed in glaucophytes [57]. Phylogenetic analysis showed that four enzymes in the Calvin-Benson cycle originated from cyanobacteria, but others are of eukaryotic origin, except rubisco, whose origin is different in the green and red lineage. These chimeric origins are conserved in the three lineages. This chimerism must have been established early in the chloroplast formation before the diversification of the three lineages. In other words, this is another evidence for the monophyly of the three lineages.

In contrast, Stiller [58] argued that the similarity of green and red algae (both chloroplasts and nuclear genomes) was a result of convergent evolution with a large influx of plastid DNA into nuclear genomes, and rejected a rhodophyte-green plant sister relationship. Using slowly evolving nuclear genes, Nozaki et al. [59] suggested that the three lineages are not really monophyletic, because some non-photosynthetic clades are also included in “Plantae” that they define. These two anti-monophyletic views were not identical, but presented serious concerns about “primary endosymbiosis”. Nevertheless, Nozaki et al. [59] still try to interpret their results “under the assumption of the single plastid primary endosymbiosis”. The recent identification of non-photosynthetic predators sister to red algae named *Rhodelphis* could also break the monophyly of Archaeplastida [60]. However, the majority of researchers seem to affirm that not only the chloroplasts but also the entire cells of the three lineages are monophyletic.

The three lineages, green algae/plants, red algae, and glaucophytes, are classified in a single phylum called Archaeplastida [61]. Curiously, the definition of Archaeplastida involved the notion of cyanobacterial endosymbiosis: “Photosynthetic plastid with chlorophyll *a* from an ancestral primary endosymbiosis with a cyanobacterium; plastid secondarily lost or reduced in some; usually with cellulose cell wall; flat cristae; starch storage product.” ([61], p. 420. My emphasis). In the most recent revision [62], this was slightly changed: “Photosynthetic plastid with chlorophyll type-*a* from an ancestral primary endosymbiosis with a cyanobacterium; plastid with two membranes without periplastid endoplasmic reticulum; plastid reduced in some; usually with cell wall or other extracellular covering; flat mitochondrial cristae; starch storage product (p. 35)”. That is all that the authors gave as the criteria of classification of Archaeplastida.

We should distinguish two problems here: monophyly of the three lineages of algae/plants on the one hand, and the cyanobacterial origin of their chloroplasts on the other. The common origin of the three lineages of Archaeplastida (with or without non-photosynthetic clades) does not imply that their chloroplasts are cyanobacterial descendants.

### 2.6. Recent Discussions on the Mitochondrial Origin

At this point, I briefly summarize the situation in mitochondria. The postgenomic era opened a new way of understanding. About 1000 proteins were identified in the mitochondrial proteome. Large-scale phylogenetic analysis suggested that about 90% of them did not originate from α-proteobacteria, which is supposed to be the origin of the mitochondrial ribosome and mitochondrial DNA. Gray [14,15] proposed the “pre-endosymbiont hypothesis”. Pitis and Gabaldón [63] and Gabaldón [64] proposed the “pre-mitochondrial symbioses” hypothesis. Harish and Kurland [65] clearly stated: “mitochondria are not captive bacteria”. This could be a beginning of a new way of thinking of mitochondrial origin. We can imagine various scenarios to explain the situation: (1) Many genes for the future mitochondrial proteins had been acquired by the eukaryotic host before the formation of mitochondria by the acquisition of a proteobacterial endosymbiont. The old proteins changed their localization and function upon the formation of mitochondria. (2) A different form of mitochondrion was initially present in the eukaryotic cell, and then the mitochondrial genome was replaced by the one from a proteobacterium. At the same time, some more proteobacterial enzymes were introduced. Various other scenarios are also probable. Mitochondrial origin might not be a simple event of endosymbiosis, although many researchers still believe that an endosymbiosis of a proteobacterium was essential in establishing the mitochondria. We now focus on the situation in chloroplasts, which also have similar problems to re-examine.

### 2.7. Phylogenetic Analysis of Chloroplast Enzymes

**Figure 1:**
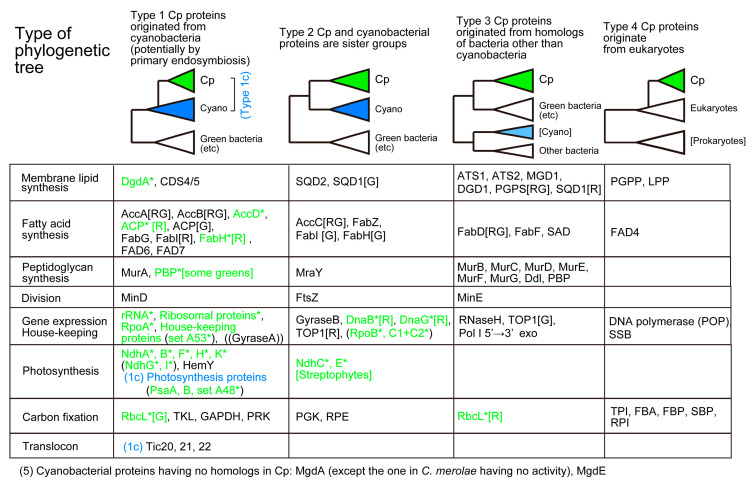
Four major types of phylogenetic relationships between cyanobacterial and chloroplast proteins. Chloroplast-encoded proteins (or RNA) are marked with an asterisk and colored. [R] and [G] indicate red and green lineages, respectively. This is an extended, revised version of the original in [9,50].

We consider here chloroplast enzymes that are encoded by both nuclear and chloroplast genomes and examine their phylogenetic relationships to the cyanobacterial homologs. Phylogenetic trees of the enzymes described in this section have been published as Supplementary Materials of a previous paper [50]. Figure 1 presents four types of phylogenetic relationships between the cyanobacterial clade and the chloroplast clade. Type 1 tree indicates that the chloroplast gene originated from cyanobacteria, which is consistent with the idea of chloroplast origin by cyanobacterial endosymbiosis. When homologs are only found in cyanobacteria and chloroplasts, we cannot determine the position of the root, and hence, the direction of evolution in a strict sense. In some special cases such as PsaA and PsaB, we can root the phylogenetic tree, but in most cases, the root position is undetermined. These cases are classified as Type 1c. In this case, *Gloeobacter* or basal *Synechococcus* are often taken as the root. In the Type 2 tree, the chloroplast clade is sister to the cyanobacterial clade. This means that the chloroplast originated from an ancestor of all extant cyanobacteria, which seems unbelievable. However, we often obtain this type of tree. This could be an artifact of phylogenetic reconstruction, but the tree form is rather robust in most cases, even if taxon samplings and phylogenetic methods are varied. We will have to find an explanation for this type of tree. Type 3 tree indicates that the chloroplast enzymes originated from bacteria other than cyanobacteria. Type 4 tree shows that eukaryotic enzymes are re-targeted to the chloroplast. Enzymes found only in cyanobacteria are classified as Type 5.

## 3. Origin of Structural Elements of Chloroplasts

### 3.1. Peptidoglycan Synthesis Enzymes

Peptidoglycan is a meshwork structure that exists between the outer and inner membranes of Gram-negative bacteria including cyanobacteria. In Gram-positive bacteria, a thick layer of peptidoglycan covers the whole cell. The presence of a wall-like layer in chloroplasts or similar photosynthetic structures was first suggested in *P. chromatophora* and *C. paradoxa* in the early 20th century. The chromatophore of *Paulinella* is now considered a structure that is phylogenetically distinct from the chloroplasts of plants and algae, but in the 1920s, it was identified as a living cyanobacterial cell within the eukaryotic cell. The organelles surrounded by the wall-like layer in *Paulinella* and *Cyanophora* were subsequently called cyanelles, meaning cyanobacterial cells living in the cell as an organelle [18] and were distinguished from the common chloroplasts. The identity of peptidoglycan in *Cyanophora* cyanelles was established by chemical analysis [66]. Peptidoglycan has long been considered important evidence for the endosymbiotic origin of not only cyanelles but also chloroplasts (see a microbiology textbook [67]. see also Item 4 of Table 1).

The presence of peptidoglycan in green plants was suggested by the genome analysis of the moss *P. patens* [68]. The complete set of enzymes involved in the synthesis of peptidoglycan was identified in the nuclear genome of *P. patens*. The presence of peptidoglycan was suggested by treatment with ampicillin, an inhibitor of peptidoglycan synthesis: namely, chloroplast division was inhibited by ampicillin in the moss. Although the role of peptidoglycan in chloroplast division is still not understood, this was taken as evidence suggesting that peptidoglycan exists in the moss chloroplast. Genes for the complete set of peptidoglycan synthesis enzymes were also found in other organisms, such as green algae (*Micromonas*), charophytes (*Klebsormidium*), liverworts (*Marchantia*), pteridophytes (*Selaginella*), and even in some gymnosperms [69]. *Arabidopsis thaliana* also possesses homologs of some of the peptidoglycan synthesis enzymes. Curiously, AtMurE was shown to be involved in chloroplast development, rather than biosynthesis of a glycan-like substance [70].

In contrast, all enzymes of peptidoglycan synthesis except MurF are encoded by the chromatophore genome of *P. chromatophora* and *Paulinella micropora*. MurF was found to be encoded by a nuclear gene, which originated from proteobacteria [9,50,71,72,73,74].

Peptidoglycan has not been observed by electron microscopy as a clearly defined layer in plant chloroplasts. Nevertheless, a well-designed experiment using the ‘Click chemistry’ was a breakthrough that identified peptidoglycan as a layer surrounding the moss chloroplast [75]. Subsequently, peptidoglycan materials were located between the outer and inner envelope membranes of moss chloroplasts by quantitative densitometry of transmission electron micrographs [76].

Then, a question is asked: “Does the presence of peptidoglycan supports the endosymbiotic origin of chloroplasts?” Recent phylogenetic analysis showed clearly that the peptidoglycan synthesis enzymes in plants and algae are monophyletic, but the majority of them originated from bacteria other than cyanobacteria [77,78]. Note that this conclusion was obtained not only by our study [77] but also by an independent study [78]. Rare examples of cyanobacteria-related enzymes include MurA and MraY (Figure 1). A class of penicillin-binding proteins (PBP) encoded by some green algal chloroplast genomes also diverged from cyanobacteria.

These results suggest that the peptidoglycan synthesis system was introduced into green algae and glaucophytes from various bacteria. However, important questions remain. First, when (or at which stage of evolution) was the peptidoglycan synthesis system introduced? The timing could be either in the common origin of Archaeplastida, or in the common ancestor of green algae and glaucophytes (if red algae diverged first), or separately in green algae and glaucophytes. In the first case, red algae must have lost the peptidoglycan synthesis system. Some *Cyanophora* enzymes are not closely related to green algal homologs and could have different origins. We also have to consider that many green algae such as *Chlamydomonas* do not have any peptidoglycan synthesis gene. We have another question: How were the genes introduced into algal cells? If each gene was introduced one by one from different bacteria, then the introduced genes could not function in peptidoglycan synthesis until all necessary genes are provided. There is no evolutionary advantage of keeping individual genes during the gene acquiring process. A possible scenario might be like this: a complete system of peptidoglycan synthesis was present at first in an organism, and each gene was replaced by horizontal gene transfer. After gene exchanges, a new complete, functional set of genes was introduced into an ancestor of algae (either of the three possibilities as above). Continued studies are necessary on this topic.

### 3.2. Lipid Biosynthesis Enzymes

Chloroplast is the major site of fatty acid synthesis in plant cells. A large proportion of fatty acids produced in the chloroplast are exported to the cytosol for the lipid synthesis in the endoplasmic reticulum (ER), while the remaining fatty acids are used for the chloroplast lipid synthesis. The proportion of the two metabolic flows depends on organisms and tissues. All the enzymes of lipid biosynthesis in the ER of plants and algae are eukaryotic enzymes that have closely related orthologs in other eukaryotes (animals and protists). To discuss the cyanobacterial origin of chloroplasts, we have to examine fatty acid synthesis and lipid synthesis within the chloroplasts.

#### 3.2.1. Fatty Acid Synthesis

Two types of fatty acid synthases (FAS) are known: Type II FAS is found in bacteria including cyanobacteria and consists of four separate subunits catalyzing the main cycle of chain elongation. Type I FAS is a large molecule containing the four enzymatic activities (plus acyl carrier protein (ACP) and other related activities) within one or two multifunctional polypeptide(s), which is found in the cytosol of fungi and animals. Fungal and animal FAS are also different in the arrangement of enzymatic active centers within the polypeptides. Because plant and algal chloroplasts contain Type II FAS, the fatty acid synthesis activity in the chloroplasts has been compared with the cyanobacterial activity. Type II FAS is also present in the mitochondria of eukaryotes including animals, plants, and other organisms, but only limited production of fatty acids is ascribed to mitochondria. Note that all enzymes involved in fatty acid synthesis (except some algal enzymes encoded by the chloroplast genome, such as red algal ACP and FabH) are encoded by the nuclear genome. Under these circumstances, fatty acid synthesis in the chloroplast was simply believed to originate from cyanobacteria. Recent phylogenetic analysis showed indeed cyanobacterial origin of many components of fatty acid synthesis, but not all (Figure 1). The condensing enzyme called FabF that catalyzes the chain elongation reaction in the chloroplast originates from green sulfur bacteria, whereas the mitochondrial homolog originates from proteobacteria [9,50]. Chloroplast FabD, which transfers malonyl CoA to ACP, is also closely related to green sulfur bacteria and green non-sulfur bacteria. The green bacteria (both sulfur and non-sulfur) are photosynthetic bacteria that do not produce oxygen but utilize light energy for carbon fixation. Curiously, in many phylogenetic analyses, green bacteria are often found to be the most related group of chloroplasts.

In the chloroplasts, the initial products of FAS are palmitic and stearic acids. In the chloroplasts of green plants and algae, stearic acid is desaturated (introduction of a double bond is called desaturation) to oleic acid by stearoyl ACP desaturase (SAD). Therefore, the primary products of fatty acid synthesis in these chloroplasts are palmitic and oleic acids. SAD is not present in red algae and glaucophytes, nor in cyanobacteria. Nevertheless, diatoms have homologs of SAD. Desaturation in cyanobacteria, including oleic acid synthesis, uses acyl lipids as the substrate rather than acyl ACP or acyl CoA.

#### 3.2.2. Chloroplast Lipid Biosynthesis

**Figure 2:**
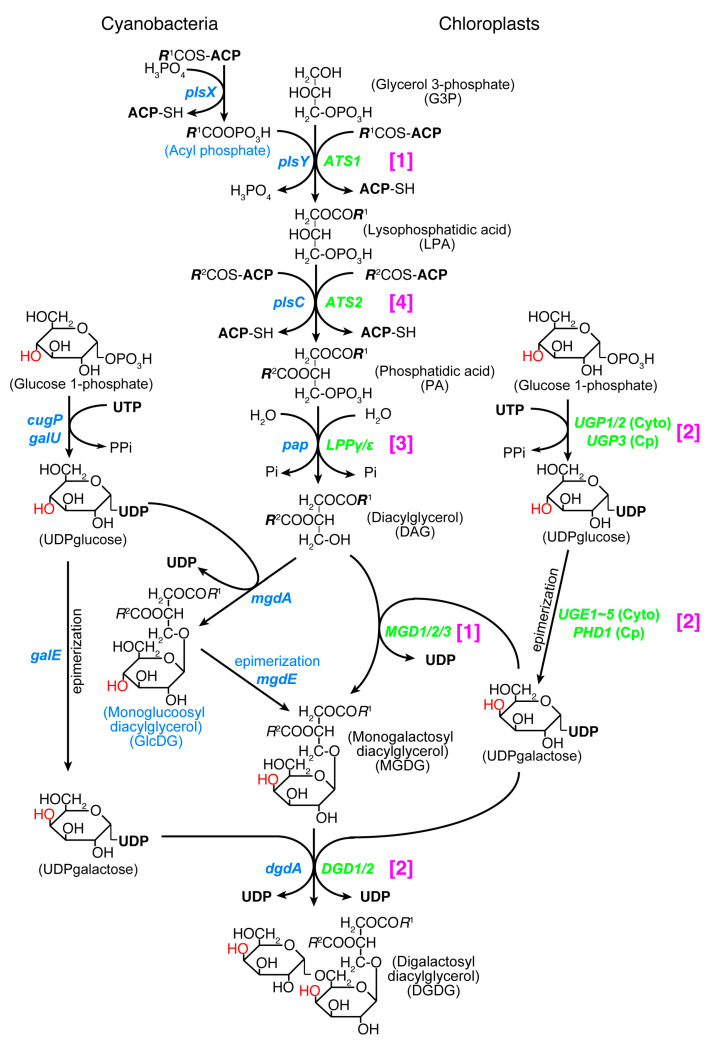
Glycolipid biosynthesis pathways in cyanobacteria and chloroplasts. The distinction between galactose and glucose is shown by the 4’-OH group in red. Cyanobacterial genes are shown in cyan, whereas eukaryotic or non-cyanobacterial genes are shown in green. Note that the biochemical reactions are different in the two steps marked [1] in magenta. In other steps, biochemically identical reactions are catalyzed by structurally unrelated (isofunctional non-homologous) enzymes marked [2]. Eukaryotic paralogs and phylogenetically distant orthologs are marked [3] and [4], respectively.

Both chloroplasts and cyanobacteria contain two galactolipids, monogalactosyl diacylglycerol (MGDG) and digalactosyl diacylglycerol (DGDG), as well as a sulfolipid, sulfoquinovosyl diacylglycerol (SQDG), and a phospholipid, phosphatidylglycerol (PG). A trace amount of phosphatidylcholine is present in the chloroplast. Tri- and tetragalactosyl diacylglycerols are also found in land plants depending on growth conditions. The set of MGDG, DGDG, and SQDG, or ‘glycolipid trio’, is indeed a common trait of oxygen-evolving photosynthetic organisms. A rare exception is *Gloeobacter* which lacks SQDG. The lack of SQDG and thylakoid membrane in this group of cyanobacteria could be related to each other, but little is known about this question. Photosynthetic bacteria without oxygen evolution also contain some of these lipids, but not the complete trio. Therefore, the glycolipid trio seemed to be good evidence for the cyanobacterial origin of chloroplasts. This prompted lipid biologists to call the lipid synthesis pathway in the chloroplast “prokaryotic pathway” [79]. This name had a connotation that the lipid biosynthetic pathway of chloroplast originated from cyanobacteria. However, this turned out to be false. The entire pathways in cyanobacteria and chloroplasts are unrelated in terms of biochemistry and phylogeny (Figure 2).

Essential differences in the galactolipid biosynthetic pathways in cyanobacteria and chloroplasts were found by labeling experiments already in the 1980s ([80,81] and the references therein). Namely, a glucolipid, monoglucosyl diacylglycerol (GlcDG), acts as an intermediate in producing MGDG in cyanobacteria. All genes encoding the galactolipid synthesis enzymes were identified in chloroplasts by the end of the 20th century [82,83], whereas the cyanobacterial genes for the synthesis of galactolipids were identified later [84,85,86,87]. The conversion of GlcDG to MGDG is an isomerization of sugar moiety called epimerization [88,89]. The enzymes involved in the synthesis of UDP-glucose and UDP-galactose, UDP-glucose pyrophosphorylase and UDP-glucose 4-epimerase, respectively, are both different in cyanobacteria and chloroplasts: UDP-glucose pyrophosphorylase is encoded by the *galU* or *cugP* genes in cyanobacteria [90], whereas it is encoded by the *UGP1/2* gene (cytoplasmic enzyme) and *UGP3* gene (chloroplast enzyme) in plants and algae (see Table 4 in [91]). These are nonhomologous, isofunctional genes. UDP-glucose 4-epimerase is encoded by the *galE* gene in cyanobacteria, while it is encoded by the *UGE1~5* genes (cytoplasmic enzyme) and *PHD1* gene (chloroplast enzyme) in plants and algae. These three kinds of genes are also nonhomologous, isofunctional genes.

Acyltransferases that produce lysophosphatidic acid and phosphatidic acid were also identified in the chloroplast in the early days, but cyanobacterial acyltransferases were identified later. *Escherichia coli* acyltransferases encoded by the *plsB* and *plsC* genes were found in the 1970s, but the acyltransferase for the first acylation was identified quite recently in other bacteria including cyanobacteria. The bacterial system uses acyl phosphate as the acyl donor of the first acylation step and two genes named *plsX* and *plsY* were identified [92]. Identification and phylogenetic analysis [93] and functional characterization [94] of the cyanobacterial genes were performed recently. The cyanobacterial pathway of galactolipid synthesis is, therefore, biochemically different from the corresponding pathway in chloroplasts in at least two points (see Figure 2). The second galactosylation step is catalyzed by two different types of galactosyltransferases in cyanobacteria (DgdA) and chloroplasts (DGD1). These two enzymes belong to the CAZy GT4 family, but their domain structures are different [95]. Note that the chloroplast genome of some red algae in Cyanidiales encodes a *dgdA* gene [96].

In addition, phylogenetic analysis of all the enzymes involved in the synthesis of galactolipids revealed that none of the chloroplast enzymes originated from cyanobacteria [9,50]. In terms of biochemical reaction, the second acylation and the dephosphorylation are common in chloroplasts and cyanobacteria. Nevertheless, phylogenetic analysis showed that the chloroplast enzymes and cyanobacterial enzymes belong to distant clades. Figure 2 shows that all enzymes in the galactolipid synthesis pathway are biochemically or phylogenetically different in cyanobacteria and chloroplasts (see Figure 1 for the summary of phylogenetic analysis). They are classified into three biochemical categories and a phylogenetic category (this is shown in magenta brackets in Figure 2). (1) Different reactions in cyanobacteria and chloroplasts: PlsX + PlsY vs. ATS1, MgdA + MgdE vs. MGD1/2/3. (2) Structurally different (non-homologous, isofunctional) enzymes: CugP/GalU vs. UGP1/2/3, GalE vs. UGE1-5/PHD1, DgdA vs. DGD1/2. (3) Chloroplast enzymes are paralogs of ER enzymes: Pap vs. LPPγ/ε. Therefore, the galactolipid biosynthesis system is unrelated to cyanobacteria and chloroplasts, and this is mostly supported by evidence that does not rely on phylogenetic analysis. (4) Only the contrast PlsC vs. ATS2 was based on phylogenetic analysis that robustly supported their distant relationship.

**Figure 3:**
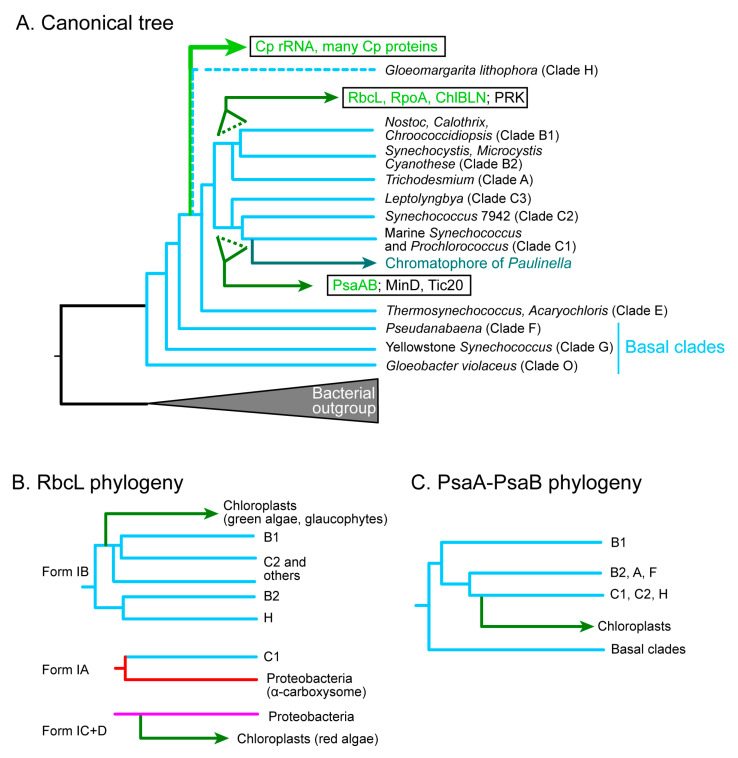
Schematic phylogenetic trees showing different origins of chloroplast enzymes (or RNA) within the cyanobacterial diversity. (**A**) Canonical phylogenetic tree of cyanobacteria and chloroplasts. Various clades of cyanobacteria are shown according to [53,54]. Chloroplast-encoded proteins and RNA are shown in green. Nuclear-encoded chloroplast proteins are shown in black. The clade names are taken from [53]. According to [54], *G. lithophora* is sister to the main chloroplast clade represented by chloroplast rRNA. For simplicity, Clade H is used for *G. lithophora*. The positions of branching of RbcL and PsaA/B are shown with dotted lines because they do not match exactly with the canonical tree. (**B**) Different origins of chloroplast and cyanobacterial RbcL. (**C**) Probable origin of PsaA and PsaB. As explained in the text and Supplementary Materials, the phylogeny of PsaA and PsaB is very difficult. This diagram, which is consistent with previous results, is still a plausible hypothesis among many other alternatives. Different line colors are used to show different lineages such as cyan for cyanobacteria and green for chloroplast. Colored protein names are used to show chloroplast-encoded proteins.

Biochemically, the biosynthetic pathways of SQDG and PG are both similar in cyanobacteria and chloroplasts, but the phylogenetic relationship of the enzymes is rather complex [50]. In SQDG synthesis, the UDP-sulfoquinovose is synthesized from UDP-glucose by SQD1/SqdB (chloroplasts/cyanobacteria, respectively [97]), and then sulfoquinovose is transferred to diacylglycerol (DAG) by SQD2/SqdX (chloroplasts/cyanobacteria, respectively [98]). SqdC replaces SqdX in some cyanobacteria. All enzymes of SQD1/SqdB are homologous, but divided into two major clades, one containing the cyanobacterial clades A-B1-B2 (see Figure 3A for the distinction between the major groups of cyanobacteria), green non-sulfur bacteria, and green algae/plants as well as glaucophytes, and another containing the cyanobacterial clades C1-C2, α-proteobacteria, and red algae (Figure 1). Chloroplast SQD2 is a single clade, sister to the cyanobacterial SqdX.

In PG synthesis, there are two different enzymes of CDP diacylglycerol synthase (CDS): the eukaryotic CDS is found in ER in plants (CDS1 and 2 in *Arabidopsis*), algae, animals, and fungi, whereas the prokaryotic CDS is found in the chloroplast (CDS4 and 5) and bacteria. Chloroplast CDS is likely to originate from cyanobacteria. This is indeed the rare case (with Cyanidiales DgdA), in which a direct phylogenetic relationship is demonstrated in cyanobacterial and chloroplast orthologs (Type 1 in Figure 1).

In summary, chloroplasts and cyanobacteria are similar in the compositions of lipids that make up thylakoid membranes, but they have essentially unrelated pathways of lipid synthesis. This could be convergent evolution, rather than vertical inheritance, that sustains the functioning of membrane-embedded components of photosynthetic machinery (photosystems, electron transfer chain, and ATP synthase). Many of the enzymes acting within the chloroplasts are eukaryotic enzymes or enzymes that are distantly related to cyanobacterial counterparts. However, many components of the chloroplast fatty acid synthesis system originate from cyanobacteria. This suggests a close relationship between photosynthesis and fatty acid synthesis, which was discussed in the context of the “fatty acid hypothesis” that explains the origin of eukaryotes [9].

## 4. Diverse Origins of Chloroplast-Encoded Genes

The chloroplast genome has been believed as a reduced cyanobacterial genome that was introduced into the hypothetical algal ancestor. Phylogenetic analysis of the rRNAs was the representative support for the cyanobacterial origin of the chloroplast. However, as pointed out in Section 2.2, this was strongly affected by the genocentric view of the time. Phylogenetic analysis of chloroplast genome has been traditionally performed with concatenated amino acid sequences of conserved genes [53,54] because chloroplast-encoded proteins (e.g., ribosomal proteins) are generally small. In practice, we obtain divergent phylogenetic trees for individual chloroplast ribosomal proteins, but a fairly reliable phylogenetic tree of concatenated ribosomal sequences that resembles the tree of rRNA [9,50]. In the phylogenetic trees of the chloroplast rRNA as well as the concatenated chloroplast-encoded proteins, the chloroplast clade branches from the basal clade of cyanobacteria after the clade E, but before the split of the clade A-B1-B2 and the clade C1-C2-C3 (Figure 3A) [9,34,35,53,54]. Recent work suggested that *Gloeomargarita lithophora* (assigned “clade H” in the present article) is the closest cyanobacterium to the chloroplasts [54]. This is the canonical phylogenetic tree of chloroplasts and cyanobacteria that supports the current notion of the cyanobacterial origin of chloroplasts (Figure 3A). It should be noted, however, that the unity of the chloroplast genome should not be the first assumption to make.

Each of the large proteins, such as the large subunit of rubisco, the RNA polymerase subunits, and the two large subunits of Photosystem I reaction center P700, has sufficient phylogenetic signals for constructing a reliable, individual phylogenetic tree. Examination of these individual trees suggested that the relationship between the cyanobacteria and the chloroplasts might not be straightforward as indicated by the canonical tree. The carbon-fixation enzyme, rubisco, was the first to present evidence for the multiple origins of the chloroplast genome. Let us examine the RbcL phylogeny.

The classification of the large subunit of rubisco, RbcL, is quite complicated. Tabita [99] presented a classification of bacterial and chloroplast RbcL based on biochemical and phylogenetic data. He showed that the red algal rubisco belongs to Form ID, whereas the rubisco of the green plants and algae, as well as many cyanobacteria, belong to Form IB. Form ID was closely related to Form IC in various proteobacteria (see Figure S1A). Delwiche and Palmer [100] proposed that the chloroplast *rbcL* (and *rbcS*, too) gene, which had been originally introduced by a cyanobacterial endosymbiont, was replaced by an α-proteobacterial homolog in the red lineage. This was a novel idea that the chloroplast genome can be a target of horizontal gene transfer. Non-cyanobacterial origin of chloroplast-encoded genes was also found in the phylloquinone/menaquinone biosynthesis genes (*menA, B, C, D, E, F*, *G*, and *H*) [101], which was interpreted as multiple horizontal gene transfer events in the ancestral endosymbiont and cyanobacteria.

Further phylogenetic analysis of the RbcL proteins revealed that bacterial RbcL is highly diversified and that the RbcL of some cyanobacteria, later classified as clade C1 [53], is sister to a clade including various proteobacteria containing α-carboxysomes (Figure S1. See also Supplementary Material 4 of [50]). The name “α-cyanobacteria” was used to call these cyanobacteria including *Prochlorococcus* and marine *Synechococcus*. Many other cyanobacteria contain β-carboxysomes and are called β-cyanobacteria, but these were a mixture of various clades as revealed in [53,54]. The two types of carboxysomes are biochemically different and correspond to different genomic structures [102,103].

Another complication of the RbcL phylogeny was found in the point of divergence of chloroplasts within the cyanobacterial clade. Note that the C1 clade has Form IA rubisco, which is not related to the chloroplast rubisco. Figure S1B–D,E (with different taxon sampling and different methods) show that the chloroplast clade of green plants/algae diverged from the base of the B1-C2 clade rather than the B2-H clade, although the branching pattern within the cyanobacteria was different from the canonical tree (Figure 3B).

Phylogenetic analysis of various chloroplast-encoded genes (see also Supplementary Material 4 of [50]) revealed that the *rpoA* gene (encoding the α subunit of prokaryotic RNA polymerase) as well as the *chlB, L, N* genes (encoding the light-independent protochlorophyllide oxidoreductase) originated from the base of the cyanobacterial clade A-B1-B2, whereas the *psaA* and *psaB* genes (encoding the P700 reaction center proteins of Photosystem I) originated from the base of the cyanobacterial clade C1-C2 (Figure 3C; see also [104] for *chlB, L, N*, and [105] for *psaA* and *psaB*). Phylogenetic analysis was performed to confirm these previous results on RpoA, PsaA, and PsaB. The result of RpoA was clearly confirmed (Figure S2), whereas the analysis of PsaA and PsaB turned out to be very difficult (see descriptions in Supplementary Materials). Various analyses using different taxon sampling and different phylogenetic methods gave inconsistent results. Finally, the previous result is identified as the most plausible hypothesis (Figure 3C). This is really a rare case of difficult phylogenetic analysis, and this tentative conclusion should be re-examined in further studies.

In these phylogenetic trees, only the *rpoA* tree showed a sister relationship between *G. lithophora* and chloroplasts. However, in this case, the position of *G. lithophora* was different from the position found in the canonical phylogenetic tree [54]. Therefore, the phylogenetic relationship of cyanobacterial species is variable depending on genes.

These findings as well as previous results [9,50] suggest that the chloroplast genome is not just a reduced form of a single ancestral cyanobacterial genome. Rather, it could be a chimera resulting from multiple gene transfer events from different clades of cyanobacteria. Nevertheless, we still have to consider that the phylogenetic origins of *rbcL* and *psaA/B* as well as some genes that are shown in Figure 3 could result from artifacts of phylogenetic reconstruction. As described above, the origin of chloroplast *psaA* and *psaB* will have to be studied further. Nevertheless, if we never considered a possibility that the chloroplast genome could be a chimera of different cyanobacterial genomes, this is a good occasion to verify or falsify this hypothesis. This was already challenged by a separate origin of the red algal *rbcL* gene. There could be more examples than those that I described above.

Against the data shown above, various criticisms can be raised. An alternative interpretation could be an ancient gene duplication followed by differential gene loss as in the case of *psbA* [106]. Indeed, *psaA* and *psaB* show a trace of gene duplication in cyanobacteria. Another alternative is an artifact due to phylogenetic inference. Indeed, the cyanobacterial parts of the trees of *rbcL*, *psaA*, *psaB*, and others as described above are somewhat different compared to the canonical tree. Gene trees of cyanobacteria will have to be rigorously re-examined before we can conclude a single origin of chloroplast genome within the cyanobacteria based on the concatenated tree, which averages different evolutionary histories of individual genes.

## 5. Other Chloroplast Enzymes

### 5.1. Enzymes Related to DNA Replication

Enzymes involved in the replication of the chloroplast genome are now identified as Plastid/protist organellar DNA polymerase (POP), a single polypeptide of putative viral origin. POP functions in both mitochondria and chloroplasts in all photosynthetic eukaryotes [107]. POP is also the mitochondrial replicase in most non-photosynthetic protists such as *Tetrahymena* and amoebozoa. In animals and fungi, DNA polymerase γ was identified in the early days of molecular biology, and many biochemistry textbooks still describe that the replication of the mitochondrial genome is catalyzed by DNA polymerase γ. Nevertheless, this is not valid in most eukaryotes other than animals and fungi. According to the typical endosymbiotic scenario, POP is supposed to replace the proteobacterial DNA polymerase III in the initial eukaryotic cell that engulfed the proteobacterial endosymbiont. In the lineage leading to animals (Holozoa) and fungi, POP was replaced by DNA polymerase γ. POP became also used in the chloroplasts after the formation of the initial photosynthetic alga [108]. During the evolution of eukaryotes, various proteins involved in the organellar replication were also replaced by non-cyanobacterial components [109]. As shown in Figure 1, the origins of chloroplast replication machinery and the enzymes involved in chloroplast transcription and translation are different in chloroplasts and cyanobacteria.

I proposed ‘discontinuous evolution of plastid genomic machinery’ based on a comparison of transcription factors and RNA polymerases [110], emphasizing the vast loss of prokaryotic transcription factors in the chloroplasts of green lineage upon the primary endosymbiosis if chloroplasts originated from cyanobacterial endosymbiosis. This idea was based on the “primary endosymbiosis”, which I accepted as a premise at the time. In other words, discontinuity must be defined by setting a reference to something continuous, which was supposed to be cyanobacterial endosymbiont—chloroplast continuity. In the current state of knowledge, the discontinuity notion could be replaced by diverse origins of various components of the chloroplast nucleoid.

### 5.2. Division Machinery

Reproduction by binary fission is an important similarity of chloroplasts and cyanobacteria (Item 3 in Table 1). Various prokaryotic components of division machinery are known. FtsZ, which is a tubulin-like protein that forms a ring at the site of cell division in bacteria, is also involved in chloroplast division in both green plants and red algae (for reviews, see [111,112]). The division site is determined by the dynamic interaction of MinC, MinD, and MinE in bacteria, whereas the role of MinD and MinE in chloroplast division was identified in plants [111,113]. Various eukaryotic components are also known in chloroplast division, such as ARC5 (a family of dynamin), ARC3, and PDV1 and 2. Note that some components (MinD, MinE, ARC3, and PDV1/2) are not found in red algae. Nevertheless, the molecular architecture of the chloroplast division rings was elucidated in the model red alga, *Cyanidioschyzon merolae* [112]. Phylogenetic analysis of FtsZ proteins showed that the chloroplast clades are sister to the cyanobacterial clade (Type 2 tree in Figure 1. See also Supplementary Material 7 of [50]). This point was not discussed in the past by researchers (e.g., [111]), maybe because they believed that the chloroplast FtsZ originated from cyanobacteria. FtsZ is duplicated into FtsZ1 and Z2 in green algae and plants. FtsZ3 is also present in some plants.

Chloroplast MinD (only present in glaucophytes, green algae, and plants) seemed to originate from cyanobacteria, whereas chloroplast MinE is not directly related to cyanobacterial MinE (Figure 1). MinD diverged from the root of the cyanobacterial clade C1-C2, rather than the base of cyanobacteria (Figure 3A). MinE is a small protein (about 100 amino acid residues), and the curious phylogenetic origin could result from insufficient phylogenetic signals. These findings suggest that FtsZ, MinD, and MinE, which act in cell division in cyanobacteria, were introduced into chloroplasts via divergent processes, rather than by a single event of cyanobacterial endosymbiosis. This should also be studied further in the future.

### 5.3. Carbon Fixation and Amino Acid Synthesis

The origin of the enzymes of the Calvin-Benson cycle is diverse (Figure 1). This was first noted by Martin and Schnarrenberger [114] using cDNA data available at the time. We found that the same pattern of chimerism is conserved in *A. thaliana* and *C. merolae* in the genome paper of *C. merolae* [56]. As described in Section 2.5, this was taken as evidence for the monophyly of red algae and plants. *C. paradoxa* also showed the same chimerism [57], supporting the monophyletic origin of the three lineages. Five enzymes in the chloroplast Calvin-Benson cycle are of the eukaryotic origin. Four enzymes originated from cyanobacteria, and two enzymes are sister to cyanobacteria (Type 2 tree). Nevertheless, the only enzyme encoded by the chloroplast genome, rubisco, must have been acquired by a process different from the main cyanobacterial gene transfers, either in the green lineage (from the B1-C2 clade. See Figure 3B) or in the red algae (from the C1 clade. See Figure 1).

Phylogenetic analysis of the amino acid synthesis enzymes in the chloroplast suggested that only 21 out of >100 Arabidopsis nuclear-encoded plastid enzymes involved in amino acid biosynthesis were unambiguously cyanobacteria-derived proteins [115]. Although the authors did not distinguish different types of phylogenetic relationships, my re-analysis using the Gclust database indicated that many of the enzymes described as cyanobacterial origin presented Type 2 tree (such as Fd-GOGAT, tryptophane synthase α and β subunits, etc.). For some other enzymes, the origins of the enzymes in the red and green lineages are different. More analysis in depth should be necessary, although the conclusion stating the non-cyanobacterial origin of the majority of enzymes seems to be valid.

### 5.4. Translocon Components

Translocon is the machinery that incorporates nuclear-encoded organellar proteins from the cytosol, where they are synthesized by the eukaryotic ribosomes (for a recent review, see [116]). In principle, translocon acts as the identifier of organelle that maintains the identity of a particular organelle, although some exceptional proteins use a different pathway to enter chloroplasts [117]. The structure of chloroplast translocon is being elucidated by extensive studies. An interesting similarity of chloroplast translocon and bacterial protein export system has been noted (see Figure 2 of [116]), although not all components are homologous in the two systems. An essential question remains as to how a similar mechanism can explain the reverse direction of protein transport. Translocon components are present in both the inner and outer envelope membranes of the chloroplast and are named Tic and Toc, respectively. Most of the translocon components are shared by green algae/plants, red algae, and glaucophytes, and this is taken as good evidence for the monophyly of the three lineages [56,118]. See also a detailed table on the distribution of components in plants and algae in [119]), and thus, the common origin of all chloroplasts. Curiously, Tic20, Tic21, Tic22, Toc75, and Tic236 of chloroplast translocon have cyanobacterial homologs. Toc75 and Tic236 have also bacterial homologs Omp85 (TamA) and TamB, respectively. Tic20, Tic21, and Tic22 are present essentially in cyanobacteria and chloroplasts of plants and algae, and therefore, belong to Type 1c in Figure 1. Because the reliable phylogenetic relationships of chloroplast and cyanobacterial homologs were not obtained for Tic21, Tic22, Tic236/TamB, and Toc75/Omp75, only a Tic20 tree is presented in Supplementary Materials (Figure S5). Tic20 diverged from the C1 clade (Figure 3A and Figure S5).

## 6. Essential Differences in Chloroplasts from *Paulinella* Chromatophores

### 6.1. Chloroplasts and Chromatophores

**Figure 4:**
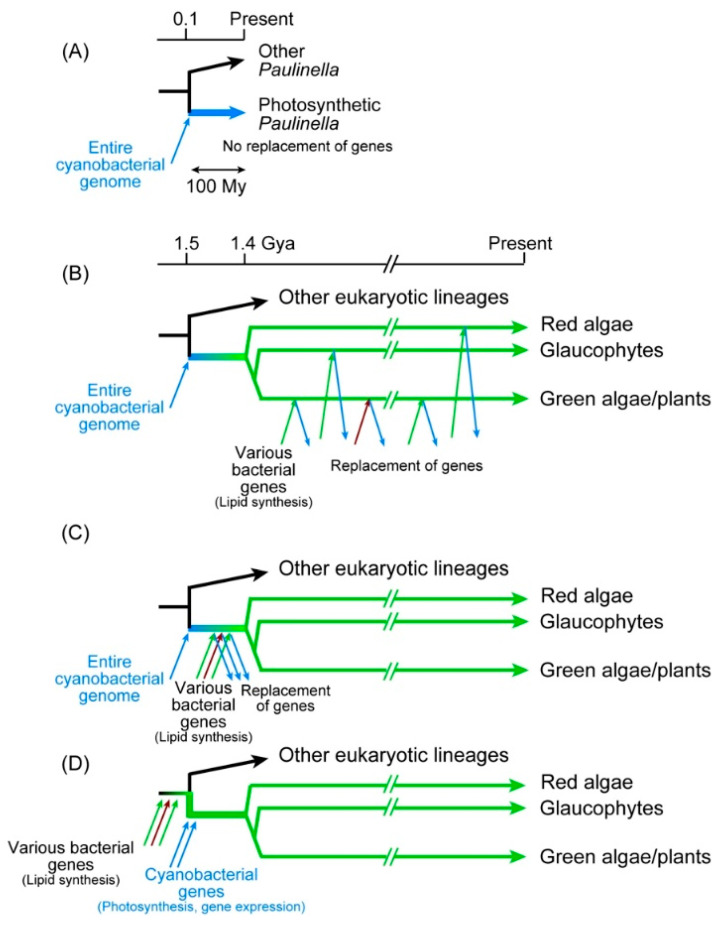
Various scenarios on the origin of non-cyanobacterial proteins in the chloroplast. (**A**) The chromatophore of *Paulinella* is a reference, in which all components originate essentially from cyanobacteria. (**B**–**D**) Various possible versions of explanations for the non-cyanobacterial proteins in chloroplasts. (**B**) Cyanobacterial proteins introduced by the primary endosymbiosis were replaced by horizontal gene transfers during the long evolution of plants and algae. (**C**) Non-cyanobacterial proteins that are conserved in the three lineages must have been introduced before the divergence of the three lineages. (**D**) Non-cyanobacterial proteins might be introduced before chloroplast formation as a pre-adaptation. Note that the time ranges of chromatophore evolution and chloroplast diversification are about the same (100 million years). See text for details. The colors are used to indicate different lineages, such as cyan for cyanobacteria and green for chloroplast.

*P. chromatophora* is a photosynthetic rhizopod having two photosynthetic organelles, called chromatophores or cyanelles [67,120,121]. As described in Section 3.1, the chromatophore genomes of *P. chromatophora* and *P. micropora*, as well as the nuclear genome of *P. chromatophora* were determined [71,72,73]. Phylogenetic analysis of the chromatophore genes revealed that the chromatophores diverged from the base of the cyanobacterial clade C1 (Figure 3A), and thus, believed to represent another case of primary endosymbiosis, or “plastid in the making” [71,122]. The relationship between chloroplasts and chromatophores has been discussed in the context of two distinct events of primary endosymbiosis [4,6,123]. The chromatophore was established about 90–140 Mya according to the estimation using 18S rRNA [124]. Therefore, the emergence of chromatophores is quite new compared to chloroplasts. The common origin of chloroplasts is estimated to be established about 1.4–1.5 Gya [125,126], indicating that chloroplasts are about 15 times older than chromatophores. This ancestry difference is often taken as the reason for the greater trait differences between the chloroplast and the cyanobacteria than those between the chromatophores and the cyanobacteria. Nevertheless, we have to recognize that the chloroplast traits common in the three lineages, green algae/plants, red algae, and glaucophytes, must have been established before the divergence of these three lineages. The time range measured from the emergence of the common ancestor of the three lineages until their divergence must have been at most about 100 million years, which is roughly similar to the ancestry of photosynthetic *Paulinella* (Figure 4).

Then, a question is asked why the chromatophore of *Paulinella* keeps its large cyanobacterial genome encoding all the enzymes of cyanobacterial lipid biosynthesis pathways. We showed by genomic and biochemical analyses that *P. chromatophora* and *P. micropora* possess authentic cyanobacterial pathways of fatty acid and lipid biosynthesis in the chromatophore, in parallel with the eukaryotic lipid biosynthetic pathway in the ER and Type I fatty acid synthase in the cytosol [74]. All the enzymes of the cyanobacterial pathway of fatty acid and lipid synthesis are encoded by the chromatophore genome, and all of them diverged from the base of the cyanobacterial clade C1 [50,93]. Therefore, the chromatophore membranes are a legacy of the cyanobacterial endosymbiont, but the situation is different in the chloroplast.

### 6.2. Possibly Different Histories of Chloroplasts and Chromatophores

Figure 4 shows possible explanations of the origin of chloroplast and chromatophore membrane lipids. The *Paulinella* system is shown in panel A. Photosynthetic *Paulinella* emerged as a result of cyanobacterial endosymbiosis about 100 million years ago. The endosymbiont genome remains as a slightly reduced form (encoding 867 and 871 proteins in *P. chromatophora* and *P. micropora*, respectively) within the chromatophore, and encodes all the enzymes of the cyanobacterial lipid biosynthesis pathways. Glycolipids (MGDG, DGDG, and SQDG) are synthesized by the enzymes encoded by the chromatophore genome.

Figure 4B–D shows various possibilities of the origin of glycolipids in chloroplasts. Many researchers simply believe scenario B (Figure 4B), in which cyanobacterial endosymbiont was introduced into the proto-algal cell, implicating that the initial system of chloroplast lipid synthesis was completely cyanobacterial. Then, during the long time of chloroplast evolution (about 1400 million years), every gene for the glycolipid synthesis was replaced by a bacterial one via horizontal gene transfer. This is the opinion of many researchers of chloroplast evolution, who consider lipid synthesis as a minor metabolic activity of the chloroplast compared to photosynthesis. However, this scenario is unlikely, because all the enzymes of glycolipid synthesis (with limited exceptions) in green algae/plants, red algae, and glaucophytes are monophyletic. Note that the rare exceptions are, as described in Section 3.2.2, CDS and red algal DgdA, the latter of which will be discussed later. The majority of the glycolipid synthesis enzymes (Figure 2) of the three lineages are monophyletic and must have already existed in the common ancestor of the three lineages (or Archaeplastida). To explain this, we have to assume that all horizontal gene transfers that provided these enzymes occurred before the divergence of the three lineages (Figure 4C). The time window in which these numerous gene transfers occurred must be within about 100 million years (note that the values 1.5 and 1.4 Gya are obtained from the molecular clock-calibrated phylogenetic trees presented in [125,126] as rough estimates). Alternatively, the cyanobacterial endosymbiont had already acquired all the glycolipid synthesis genes before the endosymbiosis. However, this is also unlikely, because the cyanobacterium (before endosymbiosis) was assumed to have two complete sets of glycolipid synthesis systems. In bacterial genetics, redundant genes are easily lost during culture. If two parallel systems existed in the ancestral endosymbiont and only a single enzyme for each reaction was selected during the subsequent chloroplast evolution, the origins of enzymes would be a mixture of cyanobacterial and non-cyanobacterial, as found in the Calvin-Benson cycle enzymes (Section 5.3). This contrasts with the situation in *Paulinella*, which did not change the lipid biosynthesis system in a comparable time range. Horizontal gene transfers could be more frequent at the time of chloroplast formation than now. However, we might be able to extend the time range before the formation of chloroplast by “primary endosymbiosis” (Figure 4D). The common ancestor of proto-alga could have already possessed the enzymes for glycolipid synthesis.

### 6.3. Extrachloroplast Glycolipids Are Traces of Their Eukaryotic Origin

The acquisition of glycolipids before chloroplast formation might seem strange for most readers, because glycolipids are components of the thylakoid membranes, and are not necessary before the beginning of photosynthesis. Nevertheless, we know that glycolipids can replace phospholipids in a condition of phosphate limitation. In *Arabidopsis*, DGDG accumulates in phosphate deficiency to replace phospholipids in various cellular membranes other than chloroplast membranes [127,128,129,130]. Even in phosphate replete conditions, accumulation of DGDG in the plasma membrane was found in the *Arabidopsis* pollen tube [131]. PG deficiency is also partially complemented by the increase of SQDG in the chloroplast [132]. Adaptive accumulation of glycolipids is also known in bacteria [133].

In the common view of plant lipid biochemists, extrachloroplast localization of glycolipids is an emergency case of re-location of chloroplast lipids accelerated by stress conditions. In animals and fungi, phospholipids are synthesized in the ER and transported to various membranes. In plants, chloroplasts and other membranes are so different in lipid composition that lipid transfers from chloroplasts to other membranes have not been considered, although lipid flow from the ER to chloroplasts is well documented. In this context, glycolipids are specific to chloroplasts, and the re-localization of glycolipids in other membranes is an exceptional phenomenon. However, the presence of glycolipids in extrachloroplast membranes in plants can be understood differently. The ability to synthesize glycolipids could be originally a eukaryotic property that is hidden behind the chloroplast glycolipids in the extant plants but becomes beneficial in particular situations such as phosphate limitation or specific cell types.

In microorganisms that do not synthesize glycolipids, phosphate limitation results in the accumulation of betaine lipids [134,135]. Betaine lipids are supposed to substitute zwitterionic phospholipids such as phosphatidylcholine and are widely distributed in algae, mosses, and pteridophytes [136]. In this respect, it is interesting to note that *P. micropora* contains a high level of betaine lipid called diacylglyceryl 3-*O*-carboxyhydroxymethylcholine (DGCC) [74]. The accidental choice of glycolipids or betaine lipids in an ancestral eukaryotic cell under phosphate starvation probably determined the fate of photosynthetic organelles in their offsprings.

### 6.4. Unified Model of Chromatophore and Chloroplast Formation

Imagine a proto-algal cell living in seawater, in which the concentration of inorganic phosphate is very low. There are two alternative lipids to overcome phosphate limitation: glycolipids or betaine lipids. If the proto-alga acquired the biosynthetic ability of glycolipids, then, glycolipids must have been beneficial to life before the beginning of photosynthesis. In this “pre-adaptation” scenario (Figure 4D), glycolipids were used to form various cellular membranes as known in extant plants. The site of glycolipid synthesis might be a small vesicle, which then, became the site of photosynthesis by acquiring various genes from cyanobacteria. We do not have to suppose acquisition of a cyanobacterial cell as a whole, but the acquisition of cyanobacterial genes is sufficient. A cyanobacterial cell could be incorporated, but the membranes were replaced by the host-derived membranes consisting of glycolipids. This is an alternative scenario for the formation of the chloroplast.

**Figure 5:**
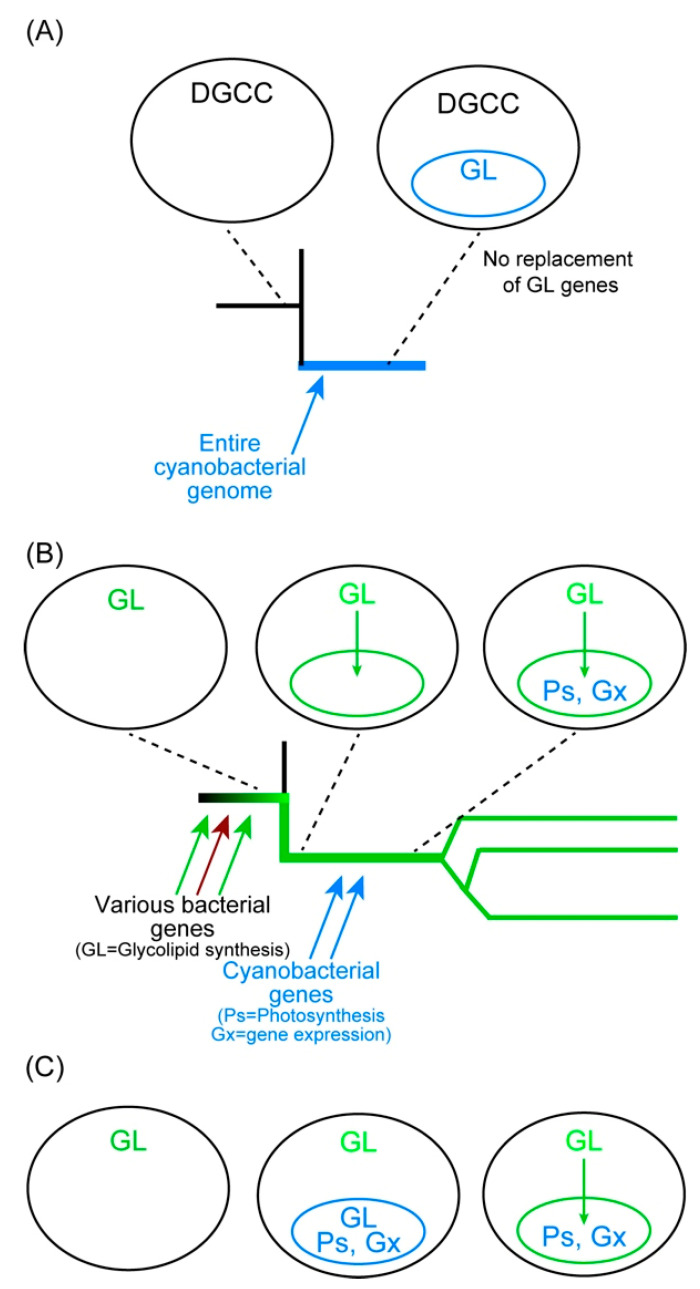
Unified schemes explaining the formation of membrane systems of (**A**) chromatophore and (**B**,**C**) chloroplast. The key components are phosphorus-free lipids. DGCC is a betaine lipid, and GL represents glycolipid trio (MGDG, DGDG, and SQDG). In scenario (**B**), glycolipids that were introduced as an adaptation to phosphate limitation form a membrane system, in which cyanobacterial photosynthesis and gene expression systems were incorporated. In scenario (**C**), the host cell acquired glycolipids before the cyanobacterial endosymbiosis, but then, the cyanobacterial glycolipid synthesis system was lost. Colors are used to indicate different lineages, such as cyan for cyanobacteria and green for chloroplast.

The case of *Paulinella* was different because betaine lipid was adopted to overcome phosphate limitation. Currently, the mechanism of DGCC synthesis is unknown, and no genes involved in its synthesis are found. Lipid analysis of non-photosynthetic *Paulinella* has not been performed. Nevertheless, we can assume that non-photosynthetic *Paulinella* has DGCC because this is a eukaryotic component of the cell that is synthesized in the ER [74]. If glycolipids but not betaine lipids are required for photosynthesis, then, *Paulinella* had to keep the complete machinery of glycolipid synthesis after the acquisition of a cyanobacterial endosymbiont which became chromatophores (Figure 5A).

In contrast, the situation of chloroplast may be viewed as Figure 5B or Figure 5C. The scenario in panel B is briefly described above. The host cell acquired glycolipid synthesis systems as an adaptation to phosphate limitation and formed a small compartment surrounded by a membrane which is the site of glycolipid synthesis. Then, various genes for photosynthesis and gene expression systems were acquired and converted the membrane compartment into a chloroplast. In a slightly different scenario in Figure 5C, a cyanobacterial cell was incorporated within the glycolipid-synthesizing compartment. At the initial stage, the whole cyanobacterial genome is kept in the compartment. The two membranes surrounding the cyanobacterial cell were soon lost, and the glycolipids necessary for the endosymbiont were provided by the host-derived membrane. Accordingly, the genes for the glycolipid synthesis enzymes were lost from the endosymbiont genome. An explanation of two membranes in the chloroplast envelope could be invagination of the original glycolipid-forming membrane at the moment of incorporation of cyanobacterial materials. This is a point to clarify in future studies.

### 6.5. Additional Notes on the Models

#### 6.5.1. DGDG Synthesis in Red Algae

The models in Figure 4 and Figure 5 are drawn with a focus on membrane lipid biosynthesis. The origins of most enzymes for the synthesis of chloroplast lipids are not cyanobacteria, but red algal DgdA should be commented on. The galactosyltransferase that produces DGDG is DgdA in cyanobacteria, which is not homologous to DGD1 in plants and algae. The chloroplast genome of Cyanidiales (or Cyanidiophytina) red algae encodes the *dgdA* gene (*ycf82*). These algae do not have the *DGD1* gene in the nuclear genome. In contrast, other red algae (Rhodophytina) have the nuclear *DGD1* gene but not the chloroplast *dgdA* gene. However, all red algae are monophyletic [137], and green algae and glaucophytes have the *DGD1* gene. As the three lineages are monophyletic, we have to assume that the ancestral red algae must have had both *DGD1* and *dgdA* genes [95]. The *dgdA* gene must have been introduced into the ancestral red algae (already possessing *DGD1*) from cyanobacteria with various other genes involved in photosynthesis and gene expression. The co-existence of *dgdA* and *DGD1* was resolved with diversification into the two lineages of red algae: The *dgdA* gene was lost in Rhodophytina, whereas the *DGD1* gene was lost in Cyanidiophytina.

#### 6.5.2. Multiple Gene Transfers from Cyanobacteria and Other Bacteria

For simplicity, the models in Figure 4 and Figure 5 are drawn with a minimal number of arrows that indicate gene transfers to the chloroplast. As explained in Section 3, gene transfers must have occurred many times, either from cyanobacteria or from other bacteria. Even the transfers from cyanobacteria occurred at least three times, from the base of the clade C1 (+C2), the base of the clades A-B1-B2, and the common base of cyanobacteria (Figure 3A). Type 2 phylogeny could be explained by gene transfer from an ancestor of cyanobacteria or a parallel, extinct lineage of cyanobacteria. Alternatively, gene transfer could occur from various bacteria to cyanobacteria (or other prokaryotes) and held within the pangenomes before being introduced into chloroplasts [138]. This could be an explanation of the diverse origins of peptidoglycan synthesis enzymes, which is otherwise difficult to explain.

#### 6.5.3. Eukaryotic Nature of Chloroplast Envelope

Microscopic observation of a plant or algal cell gives us an impression that chloroplast is a single entity, namely, a package of thylakoid membranes wrapped with envelope membranes. A chloroplast seems to correspond to a microorganism living within a eukaryotic cell. However, another view may be possible. Recent morphological studies revealed that chloroplasts are not always spherical. Many holes or invaginations were found in *Chlamydomonas* chloroplast [139,140]. Holes or pockets were also found in plant chloroplasts [141,142]. The chloroplast surface looks more like the dynamically undulating ER membranes rather than a smooth spherical plasma membrane of cyanobacteria. I propose to consider the envelope membranes as part of the eukaryotic compartment, or the interface between the eukaryotic cytosol and chloroplast stroma. In this respect, the envelope membranes could be considered as a specialized eukaryotic membrane system, in which the glycolipids are synthesized by nuclear-encoded, non-cyanobacterial enzymes (Figure 5B,C).

## 7. Conclusions: Flexible Views on the Origin

The present article focuses on the phylogenetic origin of the similarity of cyanobacteria and chloroplasts. As is well known, essentially all components of primary reactions of photosynthesis (photosystems, electron transfer, and ATP synthase) and gene expression systems (transcription and translation) of chloroplast undoubtedly originated from cyanobacteria. This led to the prevailing view of the endosymbiotic origin of chloroplasts from cyanobacteria. However, many of the common traits in cyanobacteria and chloroplasts are not attributed to common origins (Table 2). We found different biosynthetic pathways or phylogenetically unrelated enzymes in the synthesis of glycolipids and peptidoglycan. Non-cyanobacterial enzymes are also known to function in various metabolisms of chloroplasts. The currently accepted view of chloroplast origin assumes a single event of cyanobacterial endosymbiosis, probably a cyanobacterium related to *Gloeomargarita* [54], which is assumed as the common ancestor of the chloroplasts of Archaeplastida (green algae/plants, red algae, and glaucophytes). However, the single cyanobacterial endosymbiosis and the monophyly of the three lineages are different problems. Monophyly of the three lineages just supposes the common event of chloroplast formation, which could be more complicated than a single event of cyanobacterial endosymbiosis. We found at least three gene flows from cyanobacteria to the common ancestor of chloroplasts. This plurality of gene flow, as well as non-cyanobacterial enzymes involved in the traits shared by cyanobacteria and chloroplasts, suggests complexity in chloroplast formation. Biosynthesis of glycolipids could have preceded chloroplast formation as an adaptation to phosphate limitation, and it could have prepared the formation of membranes made of glycolipids, which then facilitated the incorporation of photosynthetic machinery from cyanobacteria (pre-adaptation). I call this “host-directed chloroplast formation” hypothesis [9]. This may be qualified as a “quasi-autogenous” model of chloroplast origin, but I prefer to characterize it in terms of “pre-adaptation”, which can explain the formation of both chloroplasts and chromatophores. For many researchers working on chloroplasts and cyanobacteria, the origin of membrane lipids might not be a central question. But I believe that lipid origin is crucial in all illustrations of chloroplast origin.

## Figures and Tables

**Table 1:** Evidence for the cyanobacterial origin of chloroplasts.

Item	Evidence	Explanation
1	Similarity of photosynthesis	(a) Photosynthesis with oxygen evolution (b) Presence of chlorophyll *a* and carotenoids (c) Similarity in the structure consisting of two limiting membranes and internal thylakoid membranes containing galactolipids
2	Similarity of the genetic system	Prokaryotic RNA polymerase and ribosome
3	Similarity in reproduction	Binary fission
4	Presence of peptidoglycan	Glaucophytes, some green algae, charophytes, bryophytes, pteridophytes, some gymnosperms
5	Phylogenetic relationship	(a) rRNA genes (b) Ribosomal proteins and house-keeping enzymes (c) Photosynthetic components (orthologs found only in cyanobacteria and chloroplasts)
6	Synteny of gene clusters	(a) Ribosomal proteins (b) ATPase subunits (c) RNA polymerase subunits

**Table 2:** The similarity of chloroplasts and cyanobacteria turned out to be explained by different mechanisms.

Apparent Similarity	Revealed Difference	Cyanobacteria	Chloroplasts	Reference
Chlorophylls and tetrapyrroles	Partially different biosynthesis pathway (PPO) ^1^	HemJ (or HemY)	HemY	[143]
Galactolipids (MGDG and DGDG) ^2^	Different biosynthesis pathway	MgdA- > MgdE- > DgdA	MGD1- > DGD1	[50]
Peptidoglycan ^3^	Different origins of biosynthesis enzymes	Cyanobacterial enzymes	Most enzymes originated from bacteria other than cyanobacteria	[77,78]
DNA replication	Different enzyme	PolIII	POP ^4^	[108]
Transcription	Additional enzyme in chloroplast	Prokaryotic RNAP	Prokaryotic RNAP + NEP ^5^	[110]
Division by binary fission	Additional components of division machinery in chloroplast	MinC, MinD, MinE, FtsZ, etc.	MinD, MinE, FtsZ, dynamin, etc (no MinC)	[111,112,113]
Oxygen producing photosynthesis	Partially different components of oxygen-evolving complex	PsbO, PsbU, PsbV	PsbO, PsbP, PsbQ (Red algae retain cyanobacteria-like system)	[144]

^1^ PPO, protoporphyrinogen IX oxidase. ^2^ MGDG, monogalactosyl diacylglycerol; DGDG, digalactosyl diacylglycerol. ^3^ Peptidoglycan synthesis system is found in glaucophytes, some green algae, bryophytes, pteridophytes, and some gymnosperms. ^4^ POP, plant/protist organellar DNA polymerase. ^5^ RNAP, RNA polymerase; NEP, nuclear-encoded RNA polymerase.

## Data Availability

Data are available from related publications and supplements.

## Supplementary Materials

Supplementary tables and figures are available online at https://www.mdpi.com/article/10.3390/genes12060823/s1, Table S1: Summary of phylogenetic analysis of PsaA/PsaB, Table S2: List of organisms used for the phylogenetic analysis of PsaA and PsaB, Figure S1: Phylogenetic trees of RbcL, Figure S2: Phylogenetic trees of RpoA, Figure S3: Phylogenetic trees of PsaA, Figure S4: Phylogenetic trees of PsaB, Figure S5: Phylogenetic trees of Tic20.
