# Supplementary material for: Are Cyanobacteria an Ancestor of Chloroplasts or Just One of the Gene Donors for Plants and Algae?
Source: Genes (Basel). 2021 May 27;12(6):823. doi: 10.3390/genes12060823 (PMC8227023; doi:10.3390/genes12060823)

Supplementary Materials

Are Cyanobacteria an Ancestor of Chloroplasts or Just One of the Gene Donors for Plants and Algae?

Naoki Sato

Contents

1. Methods of phylogenetic analysis

2. Description of trees

   2.1. RbcL

   2.2. RpoA

   2.3. PsaA

   2.4. PsaB

   2.5. Tic20

3. References

Table S1 Summary of phylogenetic analysis of PsaA/PsaB

Table S2 List of organisms used for the phylogenetic analysis of PsaA and PsaB

Figure S1 Phylogenetic trees of RbcL

Figure S2 Phylogenetic trees of RpoA

Figure S3 Phylogenetic trees of PsaA

Figure S4 Phylogenetic trees of PsaB

Figure S5 Phylogenetic trees of Tic20

1. Methods of phylogenetic analysis

1.1. Data

Sequence data were obtained from the pre-formed protein clusters of Gclust (http://gclust.c.u-tokyo.ac.jp/). The Gclust database consists of homolog clusters of sequenced genomes [145,146]. For the phylogenetic trees shown below, I used the dataset Cyanoclust2018 that includes 72 proteobacteria, 109 cyanobacteria, 66 other bacteria, 52 plastids and chromatophores, and 11 Archaea. Gclust clusters were prepared according to the "entropy-optimized organism count method" so that they include orthologs and very closely related homologs. Nuclear-encoded plant and algal homologs of Tic20 were obtained from the dataset Gclust2012 and added to the cyanobacterial homolog cluster. Initially, phylogenetic analysis with all homologs of all species was performed, but the phylogenetic trees presented in these supplementary materials include a limited number of species.

1.2. Phylogenetic analysis

Multiple alignments of the homolog sequences (amino acid sequences) were performed by the software Muscle version 3.8.31 [147]. The alignment was checked by visualizing with the software ClustalX version 2 [148]. To obtain the sites having phylogenetic signals, I used the "getclu" command of the software SISEQ [149]. Accordingly, the low-quality parts at both N- and C- termini, as well as the sites containing gaps in more than 20% sequences, were removed. This procedure normally gives reliable alignments as shown previously [93].

Initial phylogenetic analysis was performed by the software PHYLIP ("neighbor" command), and long branches were removed by visual inspection. Then, phylogenetic analysis (maximum likelihood method: ML) was performed by the software PhyML version 3 [150] (options were: -d aa –m LG –s BEST –b -5). LG model was chosen by the ProtTest 3 software version 3.4.2 [151]. This was consistent with previous results of various chloroplast proteins [77, 93]. Then, long branches and strange patterns (such as splitting off a phylum into several groups, etc) were checked, and the taxa causing problems were eliminated again. There is no objective way of this process. The effectiveness of the process was checked by repeated tree constructions.

Then, a new round of alignment and phylogenetic analysis was performed. If a reasonable tree (this is a very subjective impression, but this will be finally checked by the consistency of ML and BI trees) was obtained, phylogenetic analysis was also performed by the Bayesian Inference (BI) method. For BI trees, the software MrBayes version 3.2.7 [152] was used with the following parameters: lset rates=invgamma, prset aamodelpr=fixed(lg), prset ratepr=variable, and mcmc ngen= 20,000,000. samplefreq and burnin were appropriately set depending on the value of ngen. The burnin values were set to remove 30% of the generated trees. Various trials were performed to obtain convergence. If the trees of ML and BI are not consistent (at least for main branchings), putative problematic taxa were eliminated. This process was repeated several times to finally obtain consistent trees of ML and BI methods.

BI analysis was also performed with the software PhyloBayes version 4.1 [153] with the CAT + GTR model. This is hereafter called PB analysis. The CAT model is reportedly robust to long-branch attraction.

In the analysis of PsaA and PsaB, I noticed that taxon sampling and phylogenetic methods seriously affected the results of phylogenetic analysis. This will be explained in *2.3* and *2.4*.

2. Description of trees

The number preceding each taxon name shows the cluster number in the Cyanoclust2018 database. Taxa are marked with cyanobacterial clade names (Fig. 3A in the main text) according to [53,54]. In many trees, a combination of numbers on each major branch indicates branch support values (a posterior probability of BI / a confidence value of ML). For simplicity, support values are not shown for minor branches. "1.00" was shown as "1" for short. Because *Halomicronema* was not included in the previous papers [53,54], it was not easy to determine which cyanobacterial clade it belongs to. *Halomicronema* is related to *Prochlorothrix* in the C2 clade. But the actual phylogenetic data (Fig. S1, S2, and S5) showed its association to *Acaryochloris* in the E clade. The two copies of PsaA and PsaB in *Halomicronea* are diverse, and it is difficult to say which of them is the native copy. *Halomicronema* was not used in the discussions on clade association.

2.1. RbcL

Figure S1 shows phylogenetic trees of RbcL with different sets of taxa.

(A) BI tree (57 taxa, 471 sites) of RbcL with ML confidence values.

The phylogenetic tree of RbcL is a complex tree composed of different clades known as "forms". The chloroplast clade of green plants/algae plus glaucophytes is sister to a large clade of cyanobacteria (clades B1, C2, C3, E, and O) having β-carboxysomes (Form IB), which also contain *Gloeobacter* in the middle that is normally located at the root of all cyanobacteria. Note that *G. lithophora* RbcL was not sister to the green chloroplast RbcL. The root of Form IB is the Yellowstone cyanobacterium (clade G), which is another basal cyanobacterium. Many horizontal gene transfers were identified in rubisco [100]. The chloroplast clade of red algae (Form ID) has been known to originate from α-proteobacteria (Form IC). The marine species of cyanobacteria (clade C1) that contain α-carboxysomes have been known to acquire rubisco (Form IA) from bacteria containing α-carboxysomes. This tree almost correctly reconstructs the known relationships of various forms of rubisco reported in the literature [99,100], although very long branches are included. Removal of some long branches causes deformation of trees, suggesting a balance of various long branches.

(B) ML tree and (C) PB tree of RbcL (37 taxa, 475 sites).

In these trees, two sequences of bacteria containing α-carboxysomes (Form IA) are taken as an outgroup. Form IA RbcL sequences of the clade C1 cyanobacteria were not included to avoid long branches. The chloroplast clade diverged from a large clade consisting of the clades, B1, C2, C3, E, A, F and O in both

trees.

(D) BI/ML tree and (E) PB tree of RbcL (35 taxa, 474 sites).

In these trees, Form IA sequences were no longer included, and rooted with the clade G. The chloroplast clade diverged from the cyanobacterial clades, B1, C2, C3, E, and O in the BI/ML tree, whereas clades A and F were also associated as unresolved taxa in the PB tree.

The cyanobacterial RbcL seemed to be affected by complex gene transfers, and the origin of the chloroplast RbcL was difficult to assign, but all these results were consistent and suggested that the chloroplast clade (green algae/plants plus glaucophytes) diverged from the B1 and C2 clades rather than from B2 or basal clades (G and H).

2.2. RpoA

RpoA is a subunit of prokaryotic RNA polymerase encoded by the chloroplast genome. Figure S2 shows phylogenetic trees of RpoA with different taxa.

(A) BI tree of RpoA (52 taxa, 310 sites) with ML confidence values.

The chloroplast clade was sister to *G. lithophora*. These diverged from a clade consisting of the A, B1, and B2 clades. The association of *G. lithophora* to the chloroplasts is reminiscent to the situation in the rRNA tree [54], in which both diverged from the deep root of cyanobacteria. However, the RpoA tree is different from the rRNA tree.

(B) BI/ML tree and (C) PB tree of RpoA (43 taxa, 306 sites).

In both trees, the chloroplast clade plus *G. lithophora* diverged from the cyanobacterial clade consisting of A, B1, and B2. All these trees are consistent with each other, and confirm the origin of chloroplast RpoA within the A, B1, and B2 clades. The position of *G. lithophora* is exceptional.

2.3. PsaA

PsaA and PsaB are the central subunits of the Photosystem I, and they are homologous to each other.

Figure S3 shows phylogenetic trees of PsaA with different taxa obtained by different methods.

(A) ML tree, (B) BI tree, and (C) PB tree of PsaA (33 taxa, 751 sites).

(D) ML tree, (E) BI tree, and (C) PB tree of PsaA (32 taxa, 751 sites).

Phylogenetic analysis of PsaA (and PsaB) is extremely difficult, because taxon sampling and phylogenetic methods affect seriously the resultant trees. Table S1 summarizes the results of analysis. I used four major different taxon sets with or without *G. lithophora*. The list of actual set of taxa in each analysis is shown in Table S2. Some cyanobacteria such as *Calothrix* sp. PCC 7507, *Chroococcidiopsis thermalis* PCC 7203, and *Halomicronema hongdechloris* C2206 contained, respectively, two, two, and three sets of *psaA-psaB* operons. Some other cyanobacteria, such as *Nostoc* sp. PCC 7120 and *Leptolyngbya* sp. NIES 3755 contain additional single copies of *psaB*. To minimize complications, only two sets of PsaA and PsaB were used for *Halomicronema hongdechloris* C2206, and additional single copies were removed. The resulting 48 taxa

were used in the analysis "A4 and B4" (Table S1). In this section, only results of PsaA are described. The chloroplast clade diverged from the clade consisting of B1, B2, C3 and E, which is different from the previously reported results [105]. However, removing *G. lithophora* drastically changed the tree (set A4-2), and the clades C1 and C2 were sister to the chloroplast clade. Since this discrepancy could result from long-branch attraction, long branches such as land plants, *Chlamydomonas*, *Cyanidum*, *Prochlorococcus*, and *Acaryochloris* were removed (set A4e). The resulting ML and BI trees (Fig. S3A and B) were still similar to the corresponding trees in the set A4. The PB tree of the set A4e (Fig. S3C) showed that the clades C1, C2, and H were sister to the chloroplast clade. The PB tree did not change after removal of *G. lithophora* (set A4e2: Fig. S3F). Curiously, the PB trees were similar to the ML and BI trees (Fig. S3D and E) of the set A4e2 (without *G. lithophora*). However, addition of some land plant taxa (set A4f) led to discrepancy between the ML/BI trees and the PB tree. Further removal of many chloroplast taxa (set A4g) also changed the PB tree. The set A4g2 without *G. lithophora* presented consistent results with ML, BI, and PB methods, except for the position of the clade A. These results suggest that *G. lithophora* has a strange effect on the tree form. After removal of long branches, and without *G. lithophora*, all three methods gave approximately consistent results (sets A4e2 and A4g2), suggesting that the chloroplast clade diverged from the common ancestor of the cyanobacterial clades, C1, C2, and H (plus A?).

2.4. PsaB

Figure S4 shows phylogenetic trees of PsaB with different taxa obtained by different methods.

(A) BI/ML tree, and (B) PB tree of PsaB (33 taxa, 733 sites).

(C) ML tree, (D) BI tree, and (E) PB tree of PsaB (32 taxa, 733 sites).

The phylogenetic analysis of PsaB was again very difficult: different taxon sampling with different methods gave inconsistent results (Table S1). However, the set B4e2 (33 taxa without *G. lithophora*) gave compatible results with the three methods, as in the case of the set A4e2 of PsaA. The PB analysis often associated *Pseudanabaena* to the chloroplast clade in both PsaA and PsaB, but this must be an artifact. In contrast to PsaA, the presence of *G. lithophora* changed the morphology of PB trees (Fig. S4B and E), although the branch support of the chloroplast clade with the B1+A+E+F clade in Fig. S4B was low (0.32). The B1 (+A+E+F) clade was also the closest to the combined chloroplast and C1+C2 clade in the ML analysis (Fig. S4C). In any case, the analysis of the set B4e or B4e2 showed that the clade C1+C2 was the closest to the chloroplast clade. This is also consistent with the results of PsaA.

The concatenated PsaA and PsaB sequences were also analyzed (Table S1). Consistent results were obtained by the PB analysis with both AB7c and AB7d datasets, although the branching was not completely resolved in the latter. The sister relationship between the clade C1+C2 and the chloroplast clade was also supported by the ML analysis of the set AB7c and by the BI analysis of the set AB7d.

In all analyses of PsaA and PsaB, the two clades B1 and B2 were not associated as in the canonical trees of cyanobacteria reported previously [53,54]. The split of B1 and B2 was also found in the

RbcL analysis (section *2.1*). Horizontal gene transfers must be more often within the cyanobacterial genomes than we unconsciously presume.

2.5. Tic20

Figure S5 shows phylogenetic trees of Tic20 with different taxa by different methods.

(A) BI tree of Tic 20/Ycf60 with ML confidence values (70 taxa, 155 sites).

(B) BI/ML tree of Tic20/Ycf60 (41 taxa, 158 sites).

(C) BI/ML tree of Tic20 (32 taxa, 158 sites).

The chloroplast clade is duplicated, and one of them (Ycf60) is encoded by the chloroplast genome in red algae. All other plant/algal Tic20 homologs are encoded by the nuclear genome. Archaeal homologs of red algal Ycf60 were also found. The chloroplast clade was sister to the C1 clade in all phylogenetic trees. Note that PB analysis was also attempted, but the results did not converge probably because of the small size of the protein.

3. References added in supplementary materials

145. Sato, N. Gclust: *trans*-kingdom classification of proteins using automatic individual threshold setting. *Bioinformatics* **2009**, 25, 599–605. doi: 10.1093/bioinformatics/btp047.

146. Sato, N.; Obayashi, T. Lipid pathway databases with a focus on algae. In *Methods in Molecular Biology: Plant Lipids,* vol. 2295; Bartels, D.; Dörmann, P. Eds.; Springer Science: **2021**. (in press). doi: 10.1007/978-1-0716-1362-7_26.

147. Edgar, R. C. MUSCLE: multiple sequence alignment with high accuracy and high throughput. *Nucleic Acids Res.* **2004**, 32, 1792–1797. doi: 10.1093/nar/gkh340.

148. Larkin, M. A.; Blackshields, G.; Brown, N. P.; Chenna, R.; McGettigan, P. A.; McWilliam, H.; Valentin, F.; Wallace, I. M.; Wilm, A.; Lopez, R.; Thompson, J. D.; Gibson, T. J.; Higgins, D. G. Clustal W and Clustal X version 2.0. *Bioinformatics* **2007**, 23, 2947–2948. doi: 10.1093/bioinformatics/btm404.

149. Sato, N. SISEQ: Manipulation of multiple sequence and large database files for common platforms. *Bioinformatics* **2000**, 16, 180–181. doi: 10.1093/bioinformatics/16.2.180.

150. Guindon, S.; Dufayard, J. F.; Lefort, V.; Anisimova, M.; Hordijk, W.; Gascuel, O. New algorithms and methods to estimate maximum-likelihood phylogenies: assessing the performance of PhyML 3.0. *Syst. Biol.* **2010**, 59, 307–321. doi: 10.1093/sysbio/syq010.

151. Darriba, D.; Taboada, G. L.; Doallo, R.; Posada, D. ProtTest 3: fast selection of best-fit models of protein evolution. *Bioinformatics*. **2011**, 27, 1164–1165. doi: 10.1093/bioinformatics/btr088.

152. Ronquist, F.; Teslenko, M.; van der Mark, P.; Ayres, D. L.; Darling, A.; Höhna, S.; Larget, B.; Liu, L.; Suchard, M. A.; Huelsenbeck, J. P. MrBayes 3.2: efficient Bayesian phylogenetic inference and model

choice across a large model space. *Syst. Biol.* **2012**, 61, 539–42. doi: 10.1093/sysbio/sys029.

153. Lartillot, N; Philippe, H. A Bayesian mixture model for across-site heterogeneities in the amino-acid replacement process. *Mol. Biol. Evol.* **2004**, 21, 1095–1109. doi: 10.1093/molbev/msh112.

Table S1 Summary of phylogenetic analysis of PsaA/PsaB

The closest sister clade to the plastid clade is shown. If available, the second and third closest clades are shown after slashes. Unresolved branches are shown with a comma. Glli, *Gloeomargarita lithophora* (clade H).

No.	Taxa	PhyML (LG)	MrBayes (LG)	PhyloBayes (CAT,GTR)
PsaA				
A4	48	(((B1,E),C3),B2)	(((B1,E),C3),B2)	Not analyzed
A4-2	48-Glli	(C1,C2)	(C1,C2)	Not analyzed
A4e	33	(((B1,C3),E),B2)	(((B1,C3),E),B2)	**((C1,C2),**H**)**
A4e2	33-Glli	**(C1,C2)** / A / B2	**A / (C1,C2)** / B2	**(C1,C2)**
A4f	40	(((B1,C3),A),B2)	(((B1,C3),A),B2)	((A,F),B2), ((C1,C2),H)
A4g	24	(((B1,C3),E),B2)	(((B1,C3),E),B2)	((A,F),B2)
A4g2	24-Glli	A / (C1,C2) / B2	A / (C1,C2) / B2	(C1,C2)
PsaB				
B4	48	(C1,C2)	(C1,C2)	Not analyzed
B4-2	48-Glli	(C1,C2)	(C1,C2)	Not analyzed
B4e	33	**(C1,C2)** / B2	**(C1,C2)** / B2	H / (((B1,(A,E)),F)
B4e2	33-Glli	**(C1,C2)** / (((B1,(A,E)),F)	**(C1,C2)** / B2	**(C1,C2)** / B2
B4f	40	(C1,C2) / B2	(C1,C2) / B2	H, F / ((A,B1),E)
B4g	24	H / (others unresolved)	H / (others unresolved)	H, F / (B1,(A,E))
B4g2	24-Glli	(C1,C2) / B2	(C1,C2) / B2	F / ((B1,E),A)
PsaA+B				
AB7c	45	**(C1,C2)** / B2	((B1,E),A)	**(C1,C2)**, (F,H), B2
AB7d	33	(((B1,E),A),C3)	**(C1,C2)** / B2, H, F	**(C1,C2)** / (F,H), B2

Table S2 List of organisms used for the phylogenetic analysis of PsaA and PsaB

Organism	Phylum/Clade	4	4e	4f	4g	7c	7d
**<< Chloroplasts >>**							
Arabidopsis thaliana Cp	Angiosperms	+				+	
Nicotiana tabacum (tobacco) Cp	Angiosperms	+				+	
Zea mays B73 Cp	Angiosperms	+				+	
Oryza sativa Cp	Angiosperms	+				+	
Amborella trichopoda Cp	Angiosperms	+				+	
Pinus thunbergii (pine) Cp	Gymnosperms	+		+		+	
Adiantum capillus-veneris Cp	Pteridophytes	+		+		+	
Anthoceros formosae Cp	Bryophytes	+		+		+	
Marchantia polymorpha Cp	Bryophytes	+		+		+	
Physcomitrella patens Cp	Bryophytes	+		+		+	
Klebsormidium nitens (*flaccidum*) Cp	Charophytes	+		+		+	
Mesostigma viride Cp	Streptophytes	+	+	+		+	+
Micromonas commoda RCC299 Cp	Green algae	+	+	+		+	+
Ostreococcus tauri Cp	Green algae	+	+	+		+	+
Nephroselmis olivacea Cp	Green algae	+	+	+	+	+	+
Chlamydomonas reinhardtii Cp	Green algae	+		+		+	+
Chlorella vulgaris Cp	Green algae	+	+	+		+	+
Cyanidium caldarium Cp	Red algae	+		+		+	
Cyanidioschyzon merolae Cp	Red algae	+	+	+		+	+
Galdieria sulphuraria Cp	Red algae	+	+	+		+	+
Porphyridium purpurea Cp	Red algae	+	+	+	+	+	+
Pyropia yezoensis Cp	Red algae	+	+	+		+	+
Chondrus crispus Cp	Red algae	+	+	+		+	+
Gracilaria salicornia Cp	Red algae	+	+	+		+	+
Cyanophora paradoxa Cp	Glaucophytes	+	+	+	+	+	+
**<< Cyanobacteria >>**							
Trichodesmium erythraeum IMS101	Clade A	+	+	+	+	+	+
Calothrix sp. PCC 7507 copy1	Clade B1	+	+	+	+	+	+
Calothrix sp. PCC 7507 copy2	Clade B1	+	+	+	+	+	+
Chroococcidiopsis thermalis PCC 7203 copy1	Clade B1	+	+	+	+	+	+
Chroococcidiopsis thermalis PCC 7203 copy2	Clade B1	+	+	+	+	+	+
Nostoc sp. PCC 7120	Clade B1	+	+	+	+	+	+
Cyanobacterium aponinum PCC 10605	Clade B2	+	+	+	+	+	+
Cyanothece sp. PCC 8801	Clade B2	+	+	+	+	+	+
Microcystis aeruginosa NIES 843	Clade B2	+	+	+	+	+	+
Synechocystis sp. PCC 6803	Clade B2	+	+	+	+	+	+
Prochlorococcus marinus CCMP1375	Clade C1	+					
Synechococcus sp. WH 8102	Clade C1	+	+	+	+	+	+
Paulinella sp. DL 2016a chromatophore	Rhizopods<br>Clade C1	+	+	+	+	+	+
Synechococcus elongatus PCC 7942	Clade C2	+	+	+	+	+	+
Halomicronema hongdechloris C2206 copy1	Clade C2,E?	+	+		+		
Halomicronema hongdechloris C2206 copy2	Clade C2,E?	+	+	+	+	+	+
Leptolyngbya sp. NIES 3755	Clade C3	+	+	+	+	+	+
Thermosynechococcus elongatus BP1	Clade E	+	+	+	+	+	+
Acaryochloris marina MBIC11017	Clade E	+					
Pseudanabaena sp. ABRG5_3	Clade F	+	+	+	+	+	+
*Synechococcus_* p. JA-3 3Ab	Clade G	+	+	+	+	+	+
Gloeomargarita lithophora Alchichica D10	Clade H	+	+	+	+	+	+
Gloeobacter violaceus PCC 7421	Clade O	+	+	+	+	+	+
Total taxa		48	33	40	24	45	33

Fig. S1 (Part 1)

A. BI tree of RbcL (57 taxa)

BI/ML (LG)

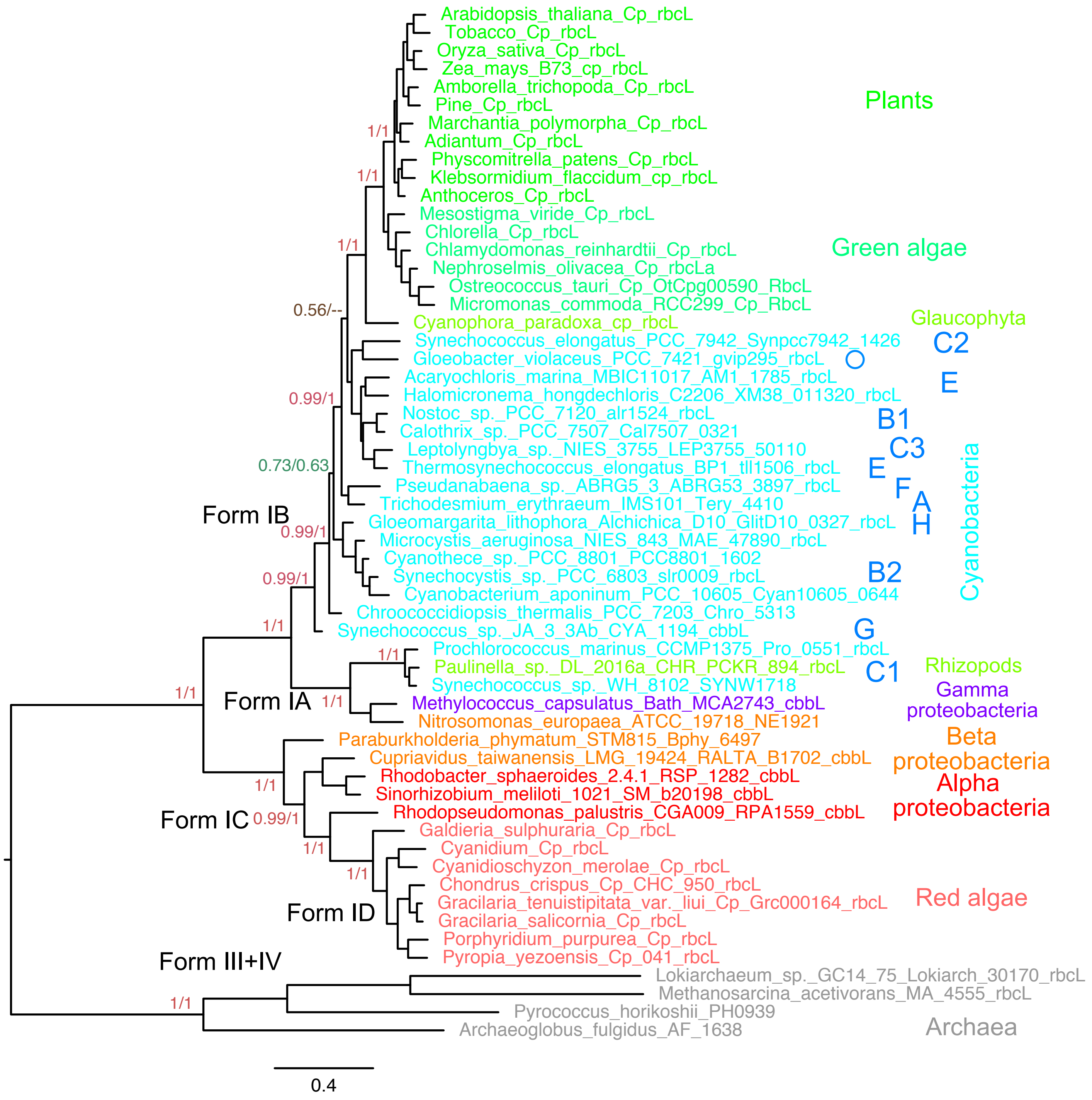

Fig. S1 (Part 2)

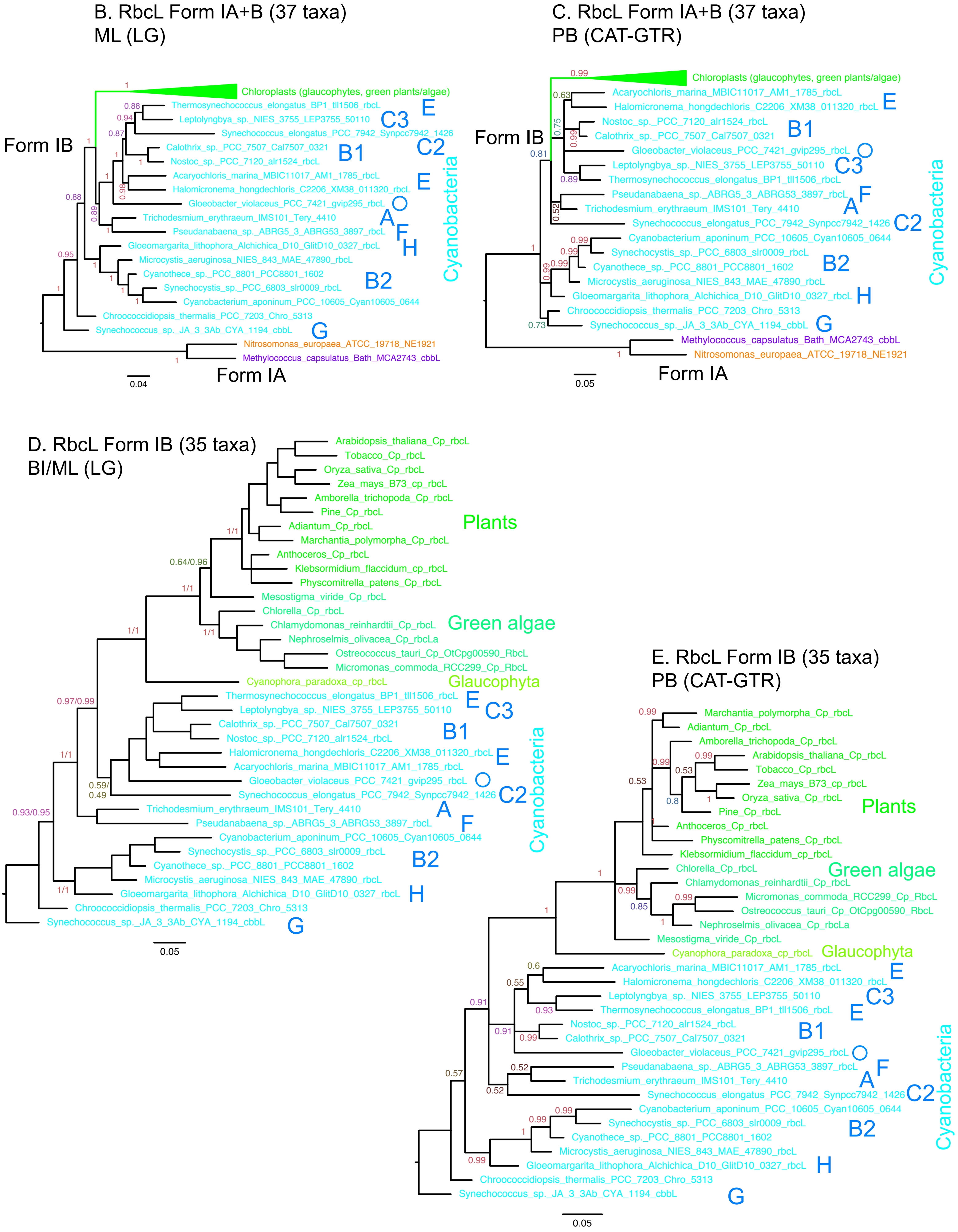

B. RbcL Form IA+B (37 taxa)
ML (LG)
Form IB
Chloroplasts (glaucophytes, green plants/algae)
Thermosynechococcus_elongatus_BP1_tll1506_rbcL
Leptolyngbya_sp._NIES_3755_LEP3755_50110
Synechococcus_elongatus_PCC_7942_Synpcc7942_1426
Calothrix_sp._PCC_7507_Cal7507_0321
Nostoc_sp._PCC_7120_alr1524_rbcL
Acaryochloris_marina_MBIC11017_AM1_1785_rbcL
Halomicronema_hongdechloris_C2206_XM38_011320_rbcL
Gloeobacter_violaceus_PCC_7421_gvip295_rbcL
Trichodesmium_erythraeum_IMS101_Tery_4410
Pseudanabaena_sp._ABRG5_3_ABRG53_3897_rbcL
Gloeomargarita_lithophora_Alchichica_D10_GlitD10_0327_rbcL
Microcystis_aeruginosa_NIES_843_MAE_47890_rbcL
Cyanothece_sp._PCC_8801_PCC8801_1602
Synechocystis_sp._PCC_6803_slr0009_rbcL
Cyanobacterium_aponinum_PCC_10605_Cyan10605_0644
Chroococcidiopsis_thermalis_PCC_7203_Chro_5313
Synechococcus_sp._JA_3_3Ab_CYA_1194_cbbL
Nitrosomonas_europaea_ATCC_19718_NE1921
Methylococcus_capsulatus_Bath_MCA2743_cbbL
Form IA
Cyanobacteria
0.04
C. RbcL Form IA+B (37 taxa)
PB (CAT-GTR)
Form IB
Chloroplasts (glaucophytes, green plants/algae)
Acaryochloris_marina_MBIC11017_AM1_1785_rbcL
Halomicronema_hongdechloris_C2206_XM38_011320_rbcL
Nostoc_sp._PCC_7120_alr1524_rbcL
Calothrix_sp._PCC_7507_Cal7507_0321
Gloeobacter_violaceus_PCC_7421_gvip295_rbcL
Leptolyngbya_sp._NIES_3755_LEP3755_50110
Thermosynechococcus_elongatus_BP1_tll1506_rbcL
Pseudanabaena_sp._ABRG5_3_ABRG53_3897_rbcL
Trichodesmium_erythraeum_IMS101_Tery_4410
Synechococcus_elongatus_PCC_7942_Synpcc7942_1426
Cyanobacterium_aponinum_PCC_10605_Cyan10605_0644
Synechocystis_sp._PCC_6803_slr0009_rbcL
Cyanothece_sp._PCC_8801_PCC8801_1602
Microcystis_aeruginosa_NIES_843_MAE_47890_rbcL
Gloeomargarita_lithophora_Alchichica_D10_GlitD10_0327_rbcL
Chroococcidiopsis_thermalis_PCC_7203_Chro_5313
Synechococcus_sp._JA_3_3Ab_CYA_1194_cbbL
Methylococcus_capsulatus_Bath_MCA2743_cbbL
Nitrosomonas_europaea_ATCC_19718_NE1921
Form IA
Cyanobacteria
0.05
D. RbcL Form IB (35 taxa)
BI/ML (LG)
Arabidopsis_thaliana_Cp_rbcL
Tobacco_Cp_rbcL
Oryza_sativa_Cp_rbcL
Zea_mays_B73_cp_rbcL
Amborella_trichopoda_Cp_rbcL
Pine_Cp_rbcL
Adiantum_Cp_rbcL
Marchantia_polymorpha_Cp_rbcL
Anthoceros_Cp_rbcL
Klebsormidium_flaccidum_cp_rbcL
Physcomitrella_patens_Cp_rbcL
Mesostigma_viride_Cp_rbcL
Chlorella_Cp_rbcL
Chlamydomonas_reinhardtii_Cp_rbcL
Nephroselmis_olivacea_Cp_rbcLa
Ostreococcus_tauri_Cp_OtCpg00590_RbcL
Micromonas_commoda_RCC299_Cp_RbcL
Cyanophora_paradoxa_cp_rbcL
Plants
Green algae
Glaucophyta
Cyanobacteria
0.05
E. RbcL Form IB (35 taxa)
PB (CAT-GTR)
Plants
Green algae
Glaucophyta
Cyanobacteria
0.05

Fig. S2 (Part 1)
A. RpoA (52 taxa)
BI/ML (LG)
With bacteria

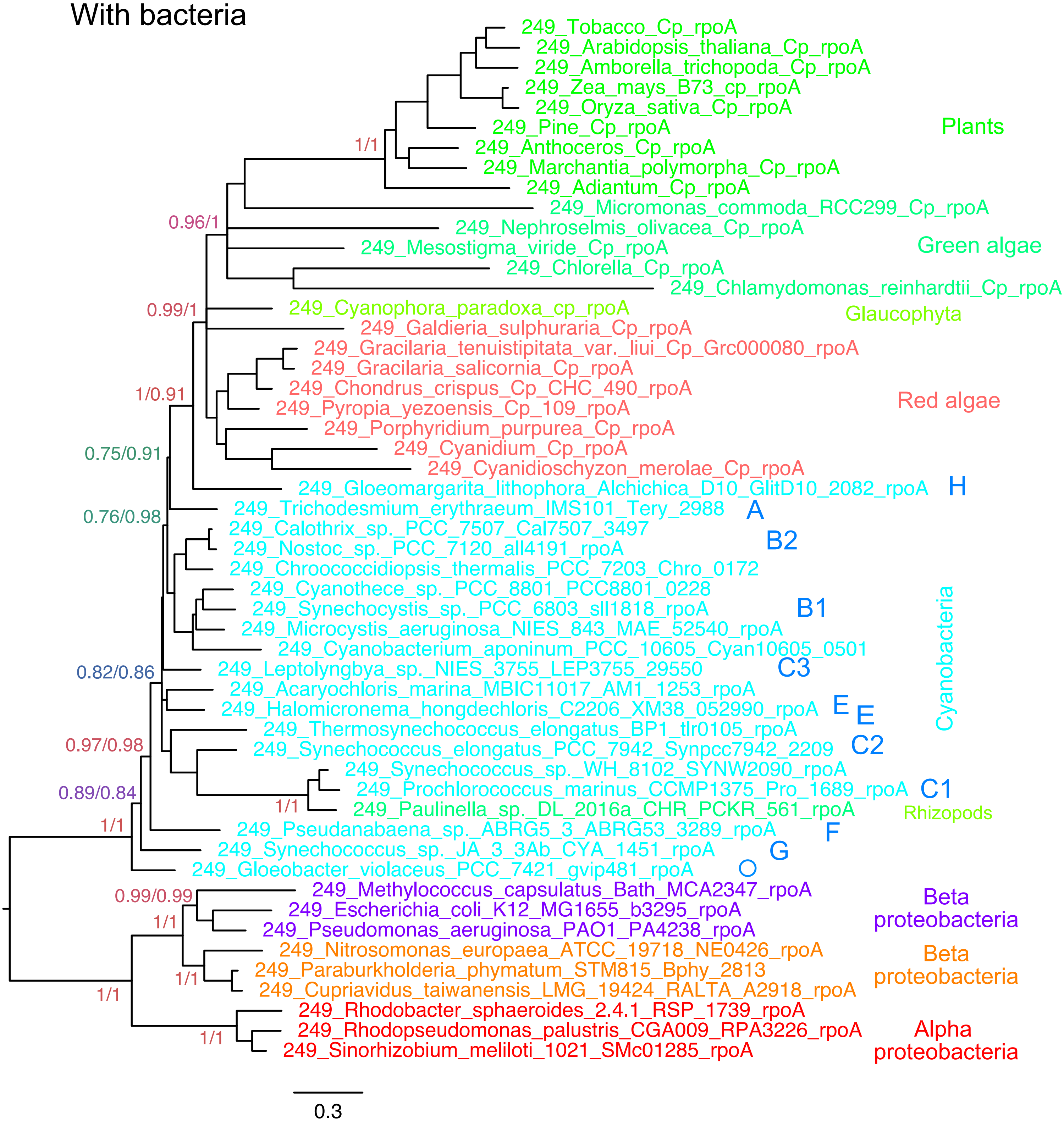

Fig. S2 (Part 2)

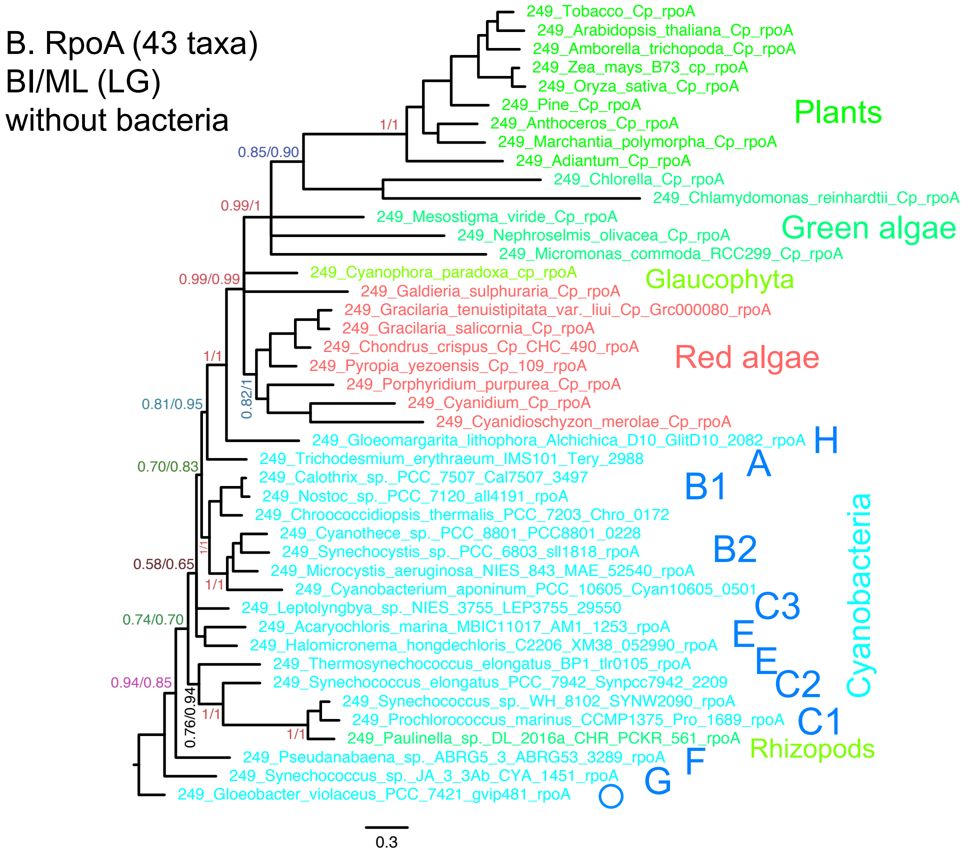
B. RpoA (43 taxa)
BI/ML (LG)
without bacteria
249_Tobacco_Cp_rpoA
249_Arabidopsis_thaliana_Cp_rpoA
249_Amborella_trichopoda_Cp_rpoA
249_Zea_mays_B73_cp_rpoA
249_Oryza_sativa_Cp_rpoA
249_Pine_Cp_rpoA
249_Anthoceros_Cp_rpoA
249_Marchantia_polymorpha_Cp_rpoA
249_Adiantum_Cp_rpoA
249_Chlorella_Cp_rpoA
249_Chlamydomonas_reinhardtii_Cp_rpoA
249_Mesostigma_viride_Cp_rpoA
249_Nephroselmis_olivacea_Cp_rpoA
249_Micromonas_commoda_RCC299_Cp_rpoA
249_Cyanophora_paradoxa_cp_rpoA
249_Galdieria_sulphuraria_Cp_rpoA
249_Gracilaria_tenuistipitata_var._liui_Cp_Grc000080_rpoA
249_Gracilaria_salicornia_Cp_rpoA
249_Chondrus_crispus_Cp_CHC_490_rpoA
249_Pyropia_yezoensis_Cp_109_rpoA
249_Porphyridium_purpurea_Cp_rpoA
249_Cyanidium_Cp_rpoA
249_Cyanidioschyzon_merolae_Cp_rpoA
249_Gloeomargarita_lithophora_Alchichica_D10_GlitD10_2082_rpoA
249_Trichodesmium_erythraeum_IMS101_Tery_2988
249_Calothrix_sp._PCC_7507_Cal7507_3497
249_Nostoc_sp._PCC_7120_all4191_rpoA
249_Chroococcidiopsis_thermalis_PCC_7203_Chro_0172
249_Cyanothece_sp._PCC_8801_PCC8801_0228
249_Synechocystis_sp._PCC_6803_sll1818_rpoA
249_Microcystis_aeruginosa_NIES_843_MAE_52540_rpoA
249_Cyanobacterium_aponinum_PCC_10605_Cyan10605_0501
249_Leptolyngbya_sp._NIES_3755_LEP3755_29550
249_Acaryochloris_marina_MBIC11017_AM1_1253_rpoA
249_Halomicronema_hongdechloris_C2206_XM38_052990_rpoA
249_Thermosynechococcus_elongatus_BP1_tlr0105_rpoA
249_Synechococcus_elongatus_PCC_7942_Synpcc7942_2209
249_Synechococcus_sp._WH_8102_SYNW2090_rpoA
249_Prochlorococcus_marinus_CCMP1375_Pro_1689_rpoA
249_Paulinella_sp._DL_2016a_CHR_PCKR_561_rpoA
249_Pseudanabaena_sp._ABRG5_3_ABRG53_3289_rpoA
249_Synechococcus_sp._JA_3_3Ab_CYA_1451_rpoA
249_Gloeobacter_violaceus_PCC_7421_gvip481_rpoA
1/1
0.85/0.90
0.99/1
0.99/0.99
1/1
0.82/1
0.81/0.95
0.70/0.83
1/1
1/1
0.58/0.65
0.74/0.70
0.94/0.85
0.76/0.94
1/1
1/1
Plants
Green algae
Glaucophyta
Red algae
H
A
B1
B2
C3
E
E
C2
C1
Rhizopods
F
G
Cyanobacteria
0.3

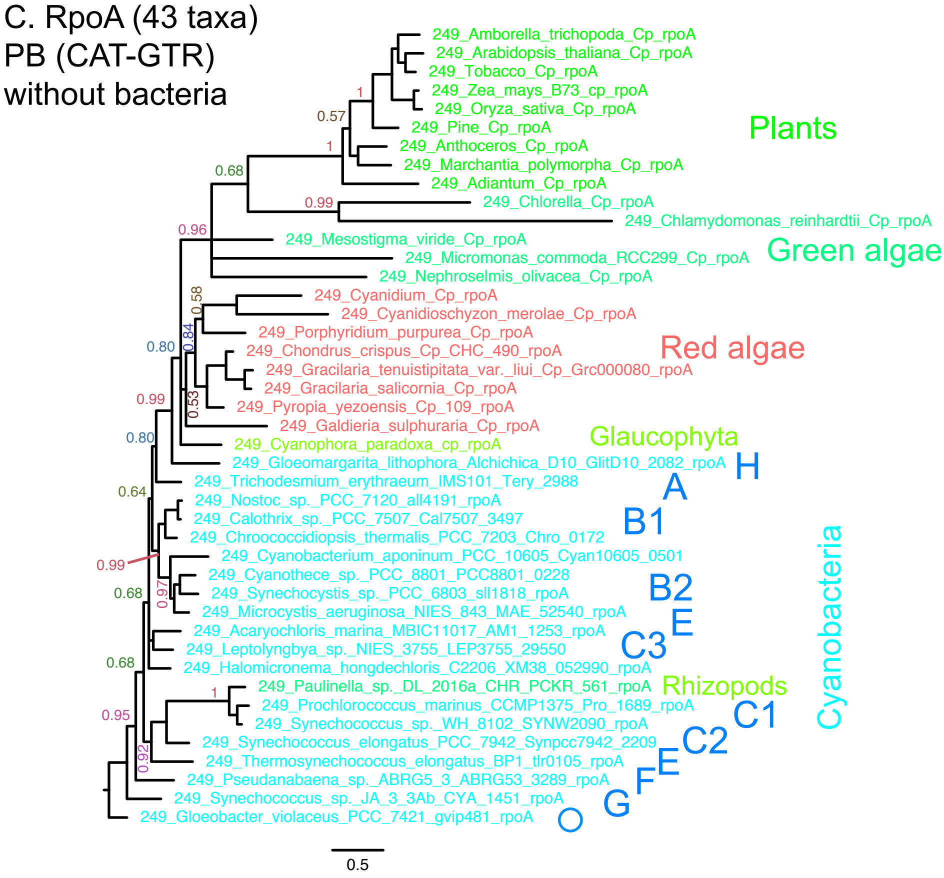
C. RpoA (43 taxa)
PB (CAT-GTR)
without bacteria
249_Amborella_trichopoda_Cp_rpoA
249_Arabidopsis_thaliana_Cp_rpoA
249_Tobacco_Cp_rpoA
249_Zea_mays_B73_cp_rpoA
249_Oryza_sativa_Cp_rpoA
249_Pine_Cp_rpoA
249_Anthoceros_Cp_rpoA
249_Marchantia_polymorpha_Cp_rpoA
249_Adiantum_Cp_rpoA
249_Chlorella_Cp_rpoA
249_Chlamydomonas_reinhardtii_Cp_rpoA
249_Mesostigma_viride_Cp_rpoA
249_Micromonas_commoda_RCC299_Cp_rpoA
249_Nephroselmis_olivacea_Cp_rpoA
249_Cyanidium_Cp_rpoA
249_Cyanidioschyzon_merolae_Cp_rpoA
249_Porphyridium_purpurea_Cp_rpoA
249_Chondrus_crispus_Cp_CHC_490_rpoA
249_Gracilaria_tenuistipitata_var._liui_Cp_Grc000080_rpoA
249_Gracilaria_salicornia_Cp_rpoA
249_Pyropia_yezoensis_Cp_109_rpoA
249_Galdieria_sulphuraria_Cp_rpoA
249_Cyanophora_paradoxa_cp_rpoA
249_Gloeomargarita_lithophora_Alchichica_D10_GlitD10_2082_rpoA
249_Trichodesmium_erythraeum_IMS101_Tery_2988
249_Nostoc_sp._PCC_7120_all4191_rpoA
249_Calothrix_sp._PCC_7507_Cal7507_3497
249_Chroococcidiopsis_thermalis_PCC_7203_Chro_0172
249_Cyanobacterium_aponinum_PCC_10605_Cyan10605_0501
249_Cyanothece_sp._PCC_8801_PCC8801_0228
249_Synechocystis_sp._PCC_6803_sll1818_rpoA
249_Microcystis_aeruginosa_NIES_843_MAE_52540_rpoA
249_Acaryochloris_marina_MBIC11017_AM1_1253_rpoA
249_Leptolyngbya_sp._NIES_3755_LEP3755_29550
249_Halomicronema_hongdechloris_C2206_XM38_052990_rpoA
249_Paulinella_sp._DL_2016a_CHR_PCKR_561_rpoA
249_Prochlorococcus_marinus_CCMP1375_Pro_1689_rpoA
249_Synechococcus_sp._WH_8102_SYNW2090_rpoA
249_Synechococcus_elongatus_PCC_7942_Synpcc7942_2209
249_Thermosynechococcus_elongatus_BP1_tlr0105_rpoA
249_Pseudanabaena_sp._ABRG5_3_ABRG53_3289_rpoA
249_Synechococcus_sp._JA_3_3Ab_CYA_1451_rpoA
249_Gloeobacter_violaceus_PCC_7421_gvip481_rpoA
1
0.57
1
0.68
0.99
0.96
0.58
0.84
0.80
0.53
0.99
0.80
0.64
0.99
0.97
0.68
0.68
1
0.95
0.92
Plants
Green algae
Red algae
Glaucophyta
H
A
B1
B2
E
C3
Rhizopods
C1
C2
E
F
G
Cyanobacteria
0.5

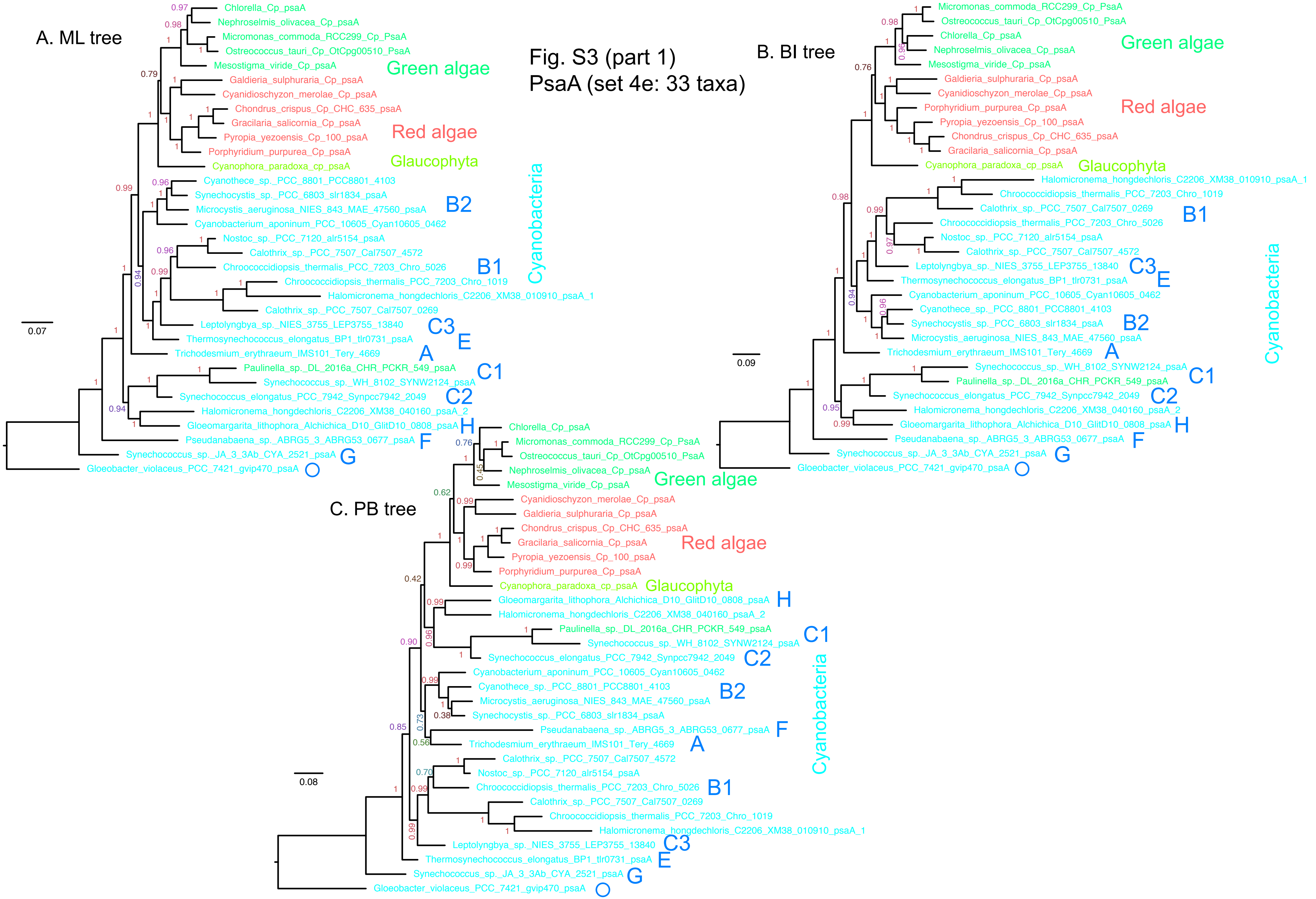

Fig. S3 (part 1)
PsaA (set 4e: 33 taxa)
A. ML tree
Chlorella_Cp_psaA
Nephroselmis_olivacea_Cp_psaA
Micromonas_commoda_RCC299_Cp_PsaA
Ostreococcus_tauri_Cp_OtCpg00510_PsaA
Mesostigma_viride_Cp_psaA
Green algae
Galdieria_sulphuraria_Cp_psaA
Cyanidioschyzon_merolae_Cp_psaA
Chondrus_crispus_Cp_CHC_635_psaA
Gracilaria_salicornia_Cp_psaA
Pyropia_yezoensis_Cp_100_psaA
Porphyridium_purpurea_Cp_psaA
Red algae
Cyanophora_paradoxa_cp_psaA
Glaucophyta
Cyanothece_sp._PCC_8801_PCC8801_4103
Synechocystis_sp._PCC_6803_slr1834_psaA
Microcystis_aeruginosa_NIES_843_MAE_47560_psaA
Cyanobacterium_aponinum_PCC_10605_Cyan10605_0462
B2
Nostoc_sp._PCC_7120_alr5154_psaA
Calothrix_sp._PCC_7507_Cal7507_4572
Chroococcidiopsis_thermalis_PCC_7203_Chro_5026
B1
Chroococcidiopsis_thermalis_PCC_7203_Chro_1019
Halomicronema_hongdechloris_C2206_XM38_010910_psaA_1
Calothrix_sp._PCC_7507_Cal7507_0269
Leptolyngbya_sp._NIES_3755_LEP3755_13840
C3
Thermosynechococcus_elongatus_BP1_tlr0731_psaA
E
Trichodesmium_erythraeum_IMS101_Tery_4669
A
Paulinella_sp._DL_2016a_CHR_PCKR_549_psaA
Synechococcus_sp._WH_8102_SYNW2124_psaA
C1
Synechococcus_elongatus_PCC_7942_Synpcc7942_2049
C2
Halomicronema_hongdechloris_C2206_XM38_040160_psaA_2
Gloeomargarita_lithophora_Alchichica_D10_GlitD10_0808_psaA
H
Pseudanabaena_sp._ABRG5_3_ABRG53_0677_psaA
F
Synechococcus_sp._JA_3_3Ab_CYA_2521_psaA
G
Gloeobacter_violaceus_PCC_7421_gvip470_psaA
O
Cyanobacteria
0.07
B. BI tree
Micromonas_commoda_RCC299_Cp_PsaA
Ostreococcus_tauri_Cp_OtCpg00510_PsaA
Chlorella_Cp_psaA
Nephroselmis_olivacea_Cp_psaA
Mesostigma_viride_Cp_psaA
Green algae
Galdieria_sulphuraria_Cp_psaA
Cyanidioschyzon_merolae_Cp_psaA
Porphyridium_purpurea_Cp_psaA
Pyropia_yezoensis_Cp_100_psaA
Chondrus_crispus_Cp_CHC_635_psaA
Gracilaria_salicornia_Cp_psaA
Red algae
Cyanophora_paradoxa_cp_psaA
Glaucophyta
Halomicronema_hongdechloris_C2206_XM38_010910_psaA_1
Chroococcidiopsis_thermalis_PCC_7203_Chro_1019
Calothrix_sp._PCC_7507_Cal7507_0269
B1
Chroococcidiopsis_thermalis_PCC_7203_Chro_5026
Nostoc_sp._PCC_7120_alr5154_psaA
Calothrix_sp._PCC_7507_Cal7507_4572
Leptolyngbya_sp._NIES_3755_LEP3755_13840
C3
Thermosynechococcus_elongatus_BP1_tlr0731_psaA
E
Cyanobacterium_aponinum_PCC_10605_Cyan10605_0462
Cyanothece_sp._PCC_8801_PCC8801_4103
Synechocystis_sp._PCC_6803_slr1834_psaA
Microcystis_aeruginosa_NIES_843_MAE_47560_psaA
B2
Trichodesmium_erythraeum_IMS101_Tery_4669
A
Synechococcus_sp._WH_8102_SYNW2124_psaA
Paulinella_sp._DL_2016a_CHR_PCKR_549_psaA
C1
Synechococcus_elongatus_PCC_7942_Synpcc7942_2049
C2
Halomicronema_hongdechloris_C2206_XM38_040160_psaA_2
Gloeomargarita_lithophora_Alchichica_D10_GlitD10_0808_psaA
H
Pseudanabaena_sp._ABRG5_3_ABRG53_0677_psaA
F
Synechococcus_sp._JA_3_3Ab_CYA_2521_psaA
G
Gloeobacter_violaceus_PCC_7421_gvip470_psaA
O
Cyanobacteria
0.09
C. PB tree
Chlorella_Cp_psaA
Micromonas_commoda_RCC299_Cp_PsaA
Ostreococcus_tauri_Cp_OtCpg00510_PsaA
Nephroselmis_olivacea_Cp_psaA
Mesostigma_viride_Cp_psaA
Green algae
Cyanidioschyzon_merolae_Cp_psaA
Galdieria_sulphuraria_Cp_psaA
Chondrus_crispus_Cp_CHC_635_psaA
Gracilaria_salicornia_Cp_psaA
Pyropia_yezoensis_Cp_100_psaA
Porphyridium_purpurea_Cp_psaA
Red algae
Cyanophora_paradoxa_cp_psaA
Glaucophyta
Gloeomargarita_lithophora_Alchichica_D10_GlitD10_0808_psaA
H
Halomicronema_hongdechloris_C2206_XM38_040160_psaA_2
Paulinella_sp._DL_2016a_CHR_PCKR_549_psaA
Synechococcus_sp._WH_8102_SYNW2124_psaA
C1
Synechococcus_elongatus_PCC_7942_Synpcc7942_2049
C2
Cyanobacterium_aponinum_PCC_10605_Cyan10605_0462
Cyanothece_sp._PCC_8801_PCC8801_4103
Microcystis_aeruginosa_NIES_843_MAE_47560_psaA
Synechocystis_sp._PCC_6803_slr1834_psaA
B2
Pseudanabaena_sp._ABRG5_3_ABRG53_0677_psaA
F
Trichodesmium_erythraeum_IMS101_Tery_4669
A
Calothrix_sp._PCC_7507_Cal7507_4572
Nostoc_sp._PCC_7120_alr5154_psaA
Chroococcidiopsis_thermalis_PCC_7203_Chro_5026
B1
Calothrix_sp._PCC_7507_Cal7507_0269
Chroococcidiopsis_thermalis_PCC_7203_Chro_1019
Halomicronema_hongdechloris_C2206_XM38_010910_psaA_1
Leptolyngbya_sp._NIES_3755_LEP3755_13840
C3
Thermosynechococcus_elongatus_BP1_tlr0731_psaA
E
Synechococcus_sp._JA_3_3Ab_CYA_2521_psaA
G
Gloeobacter_violaceus_PCC_7421_gvip470_psaA
O
Cyanobacteria
0.08

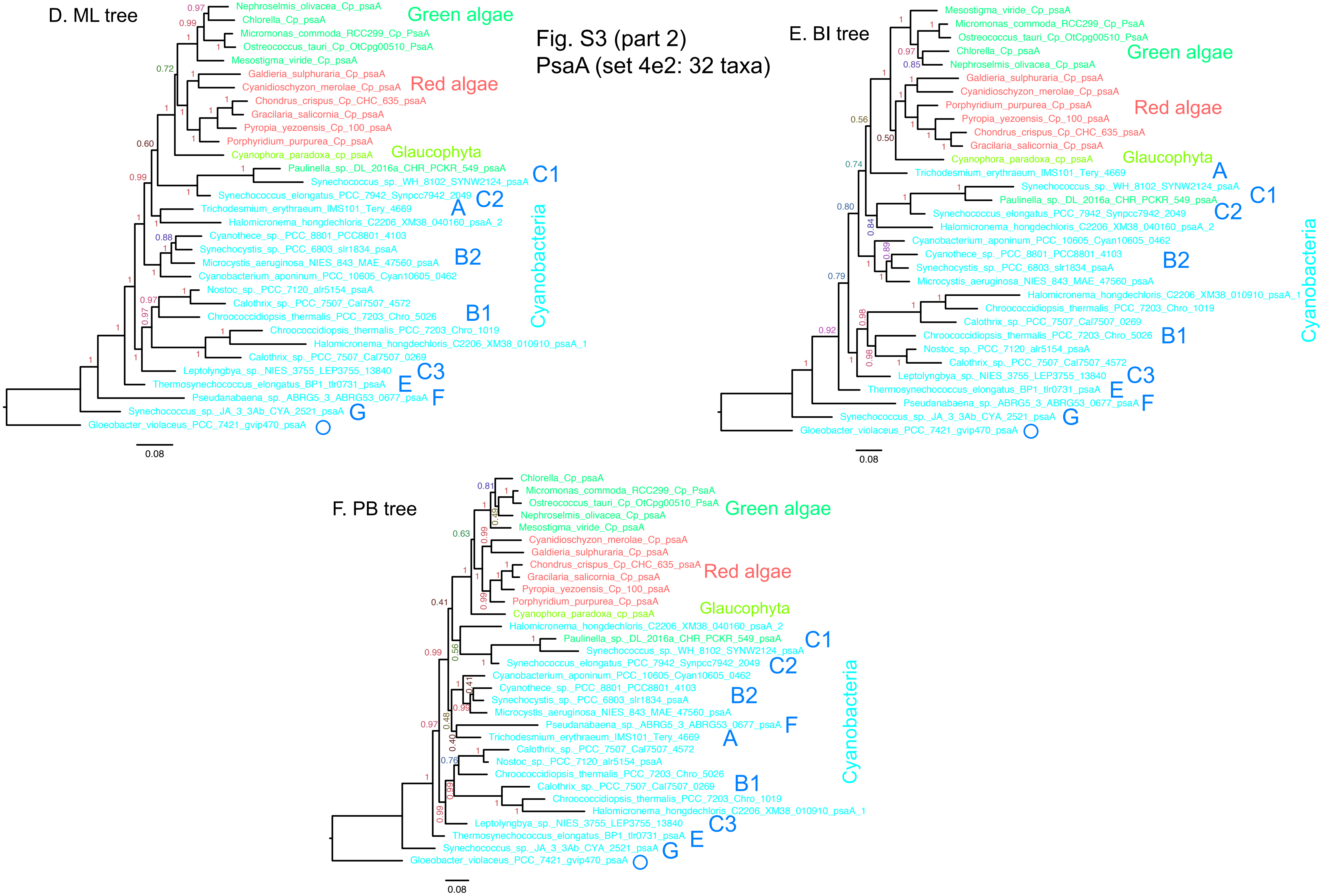

Fig. S3 (part 2)
PsaA (set 4e2: 32 taxa)

Fig. S4 (Part 1)
PsaB (set 4e: 33 taxa)

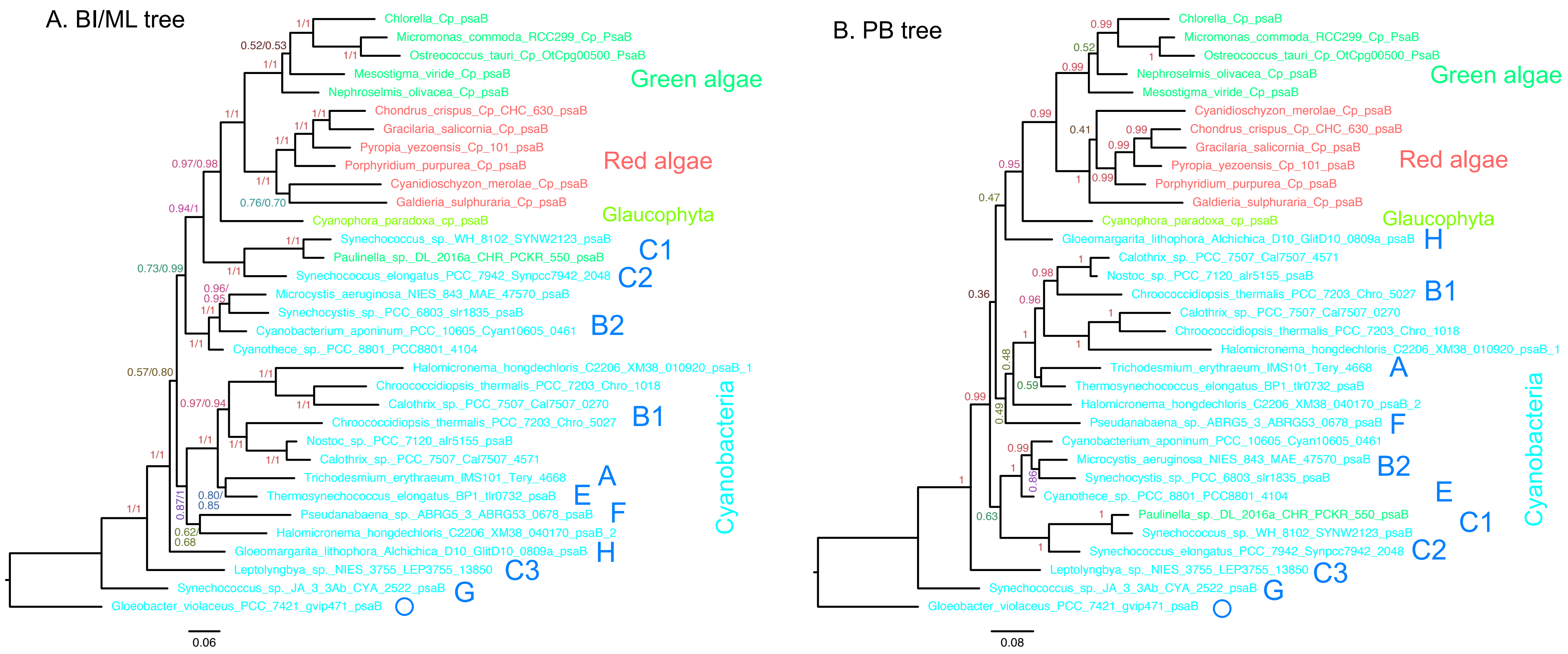

Fig. S4 (Part 2)
PsaB (set 4e2: 32 taxa)

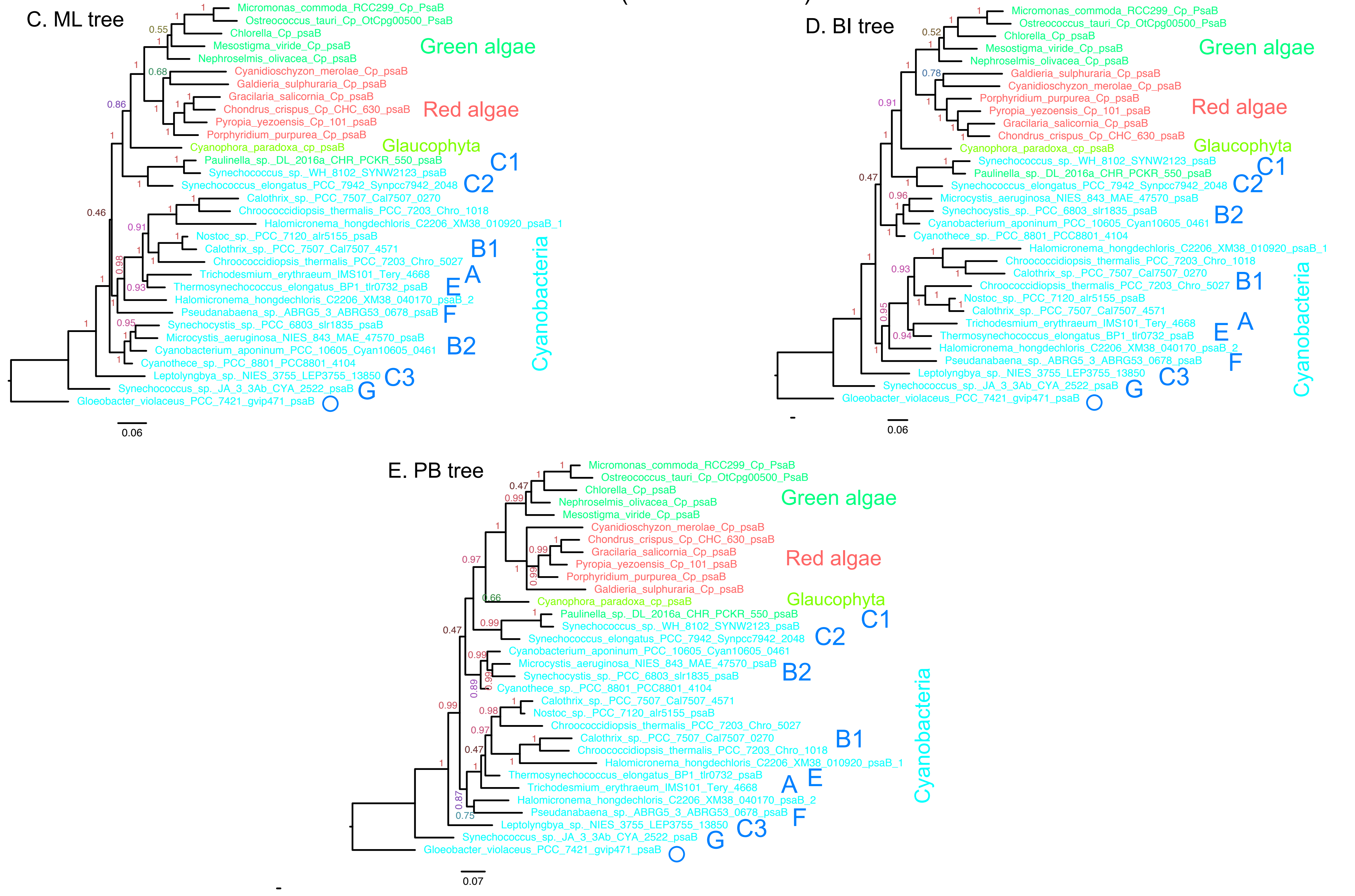

Fig. S5 A
BI tree of Tic20 (70 taxa)
BI/ML (LG)

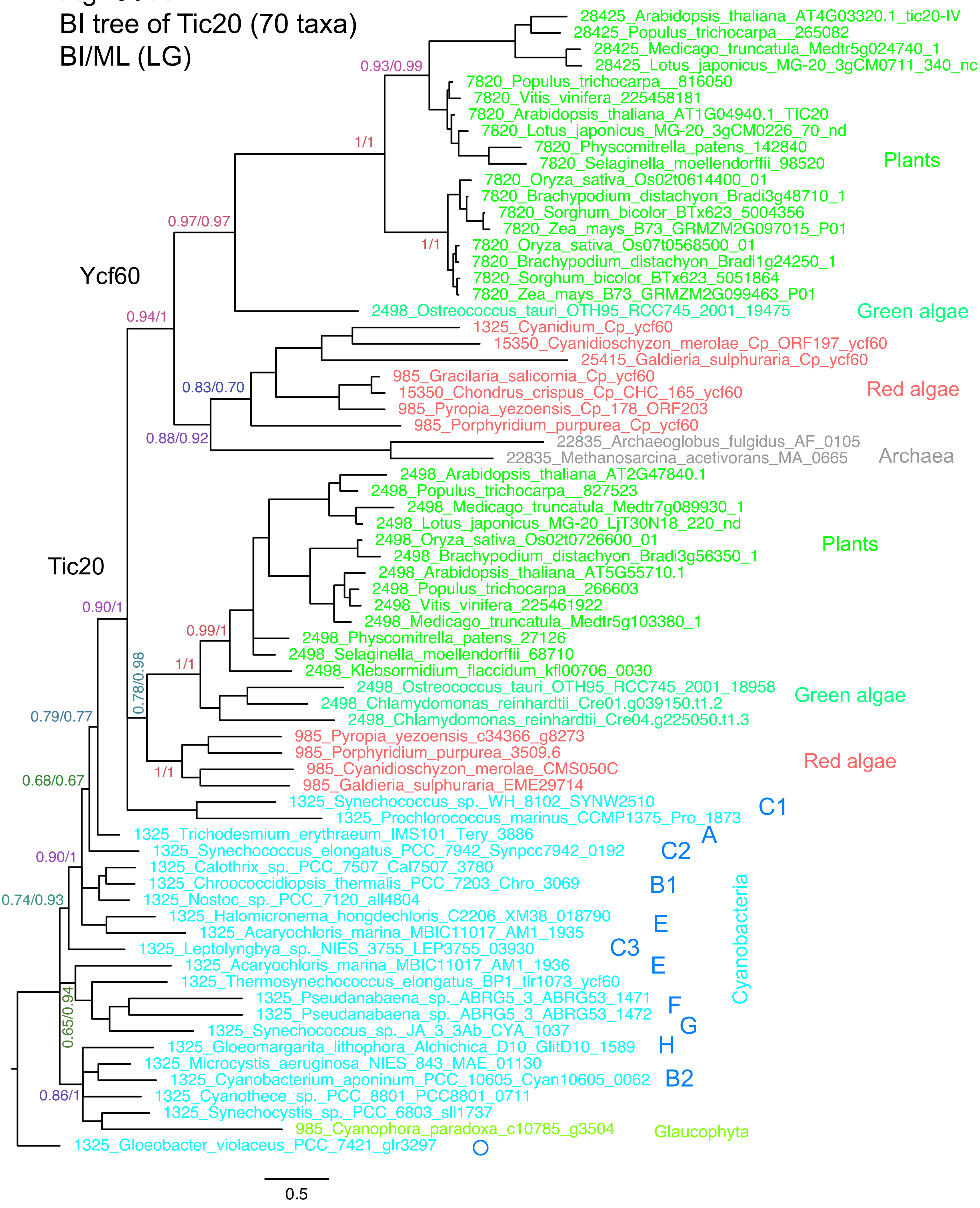

Fig. S5B
BI tree of Tic20 (41 taxa)
BI/ML (LG)

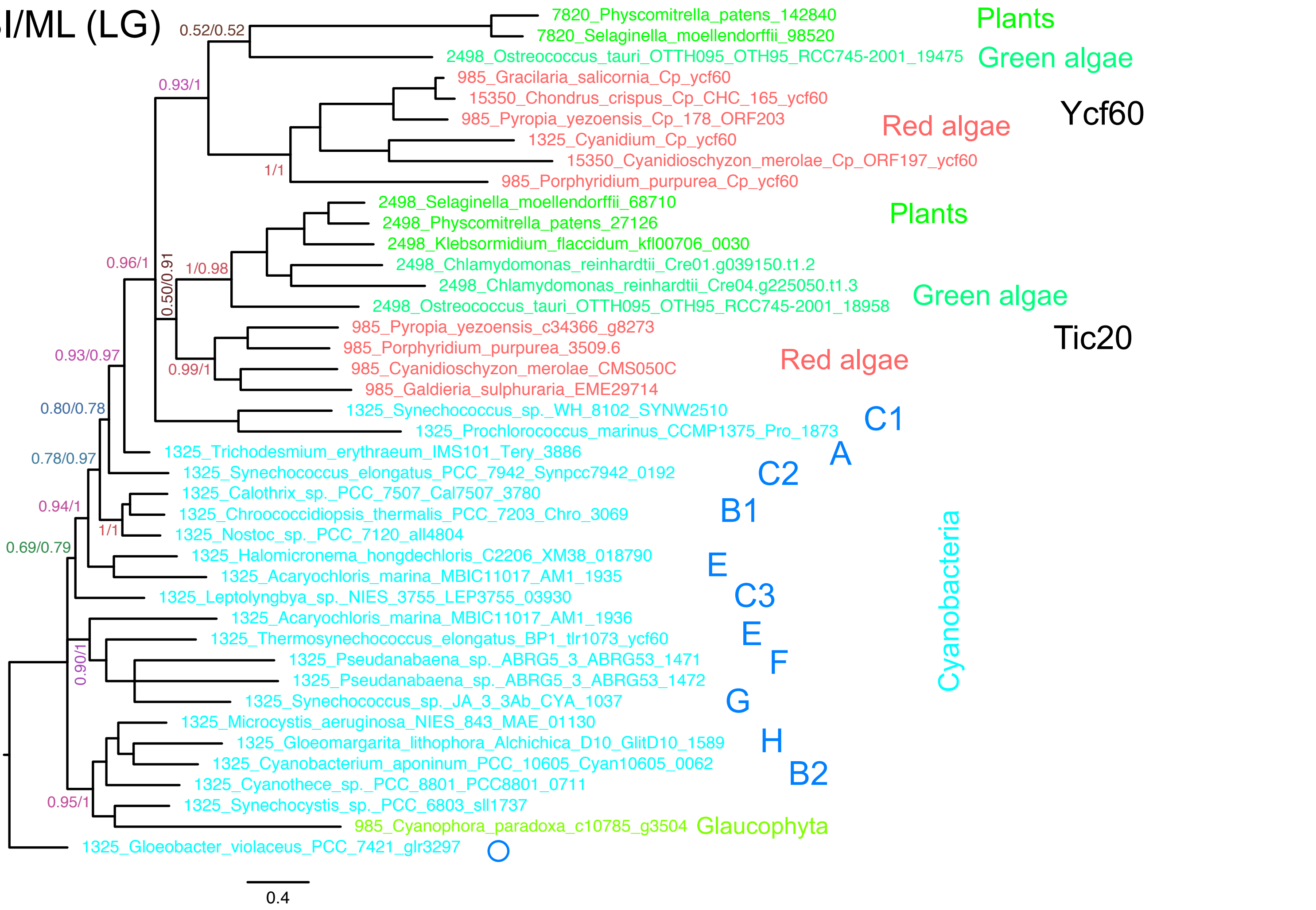

Fig. S5C
BI tree of Tic20 (32 taxa)
BI/ML (LG)

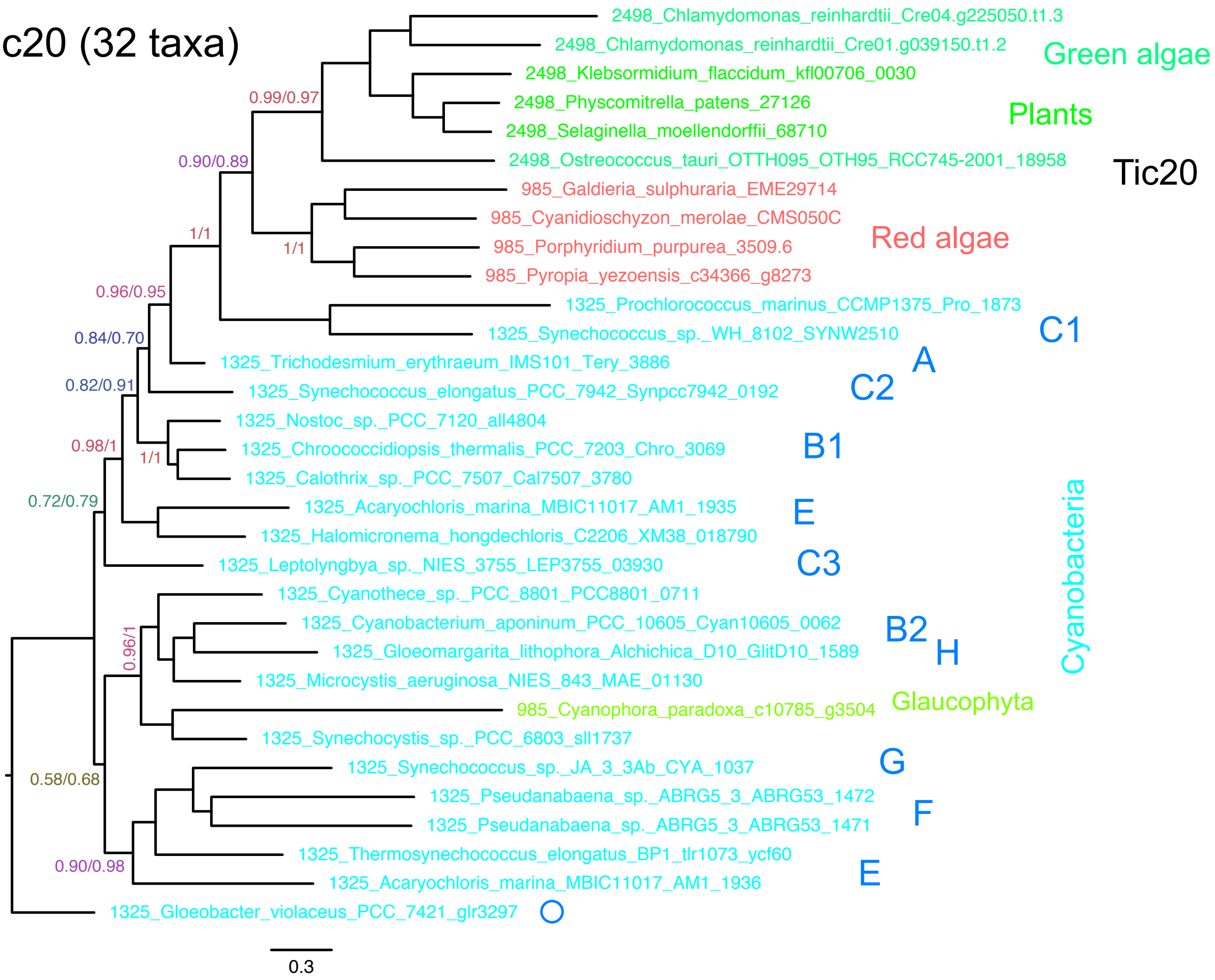

Supplement: Supplementary file 1 [file genes-12-00823-s001.zip › genes-1178906-supplementary.pdf]
